# Supplementary material for: Demographic histories and genetic diversity across pinnipeds are shaped by human exploitation, ecology and life-history
Source: Nat Commun. 2018 Nov 16;9:4836. doi: 10.1038/s41467-018-06695-z (PMC6240053; doi:10.1038/s41467-018-06695-z)
Supplement: Supplementary file 1 — Supplementary Information [file 41467_2018_6695_MOESM1_ESM.pdf]

Supplementary Information

Demographic histories and genetic diversity across pinnipeds are shaped by human exploitation, ecology and life-history

Stoffel et al.

Table of contents

Supplementary Note 1 – Figures and Tables p.3

Supplementary Note 2 – ABC analysis of postglacial expansion models p.21

Supplementary Note 3 – Genetic data collection and genotyping methods p.26

Supplementary Note 1–Figures and Tables

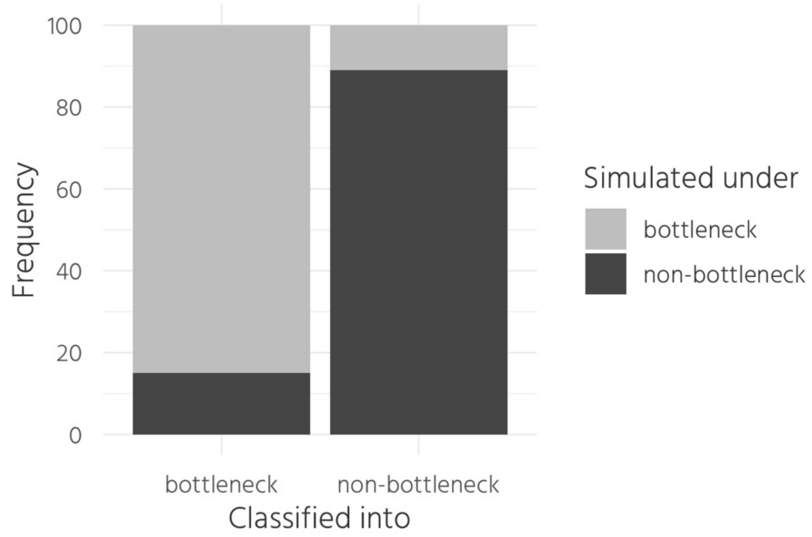

Supplementary Fig. 1: Confusion matrix plot showing the misclassification rate estimates from our model selection procedure (see Methods for details). Simulations under the bottleneck model are shown in dark grey and simulations generated under the non-bottleneck model are shown in light grey. The two bars show the classification of the simulations into either the bottleneck or the non-bottleneck model. When a simulation was randomly chosen from the bottleneck model, it was classified into the bottleneck model 85% of the time. When a simulation was randomly chosen from the non-bottleneck model, it was classified into the non-bottleneck model 89% of the time.

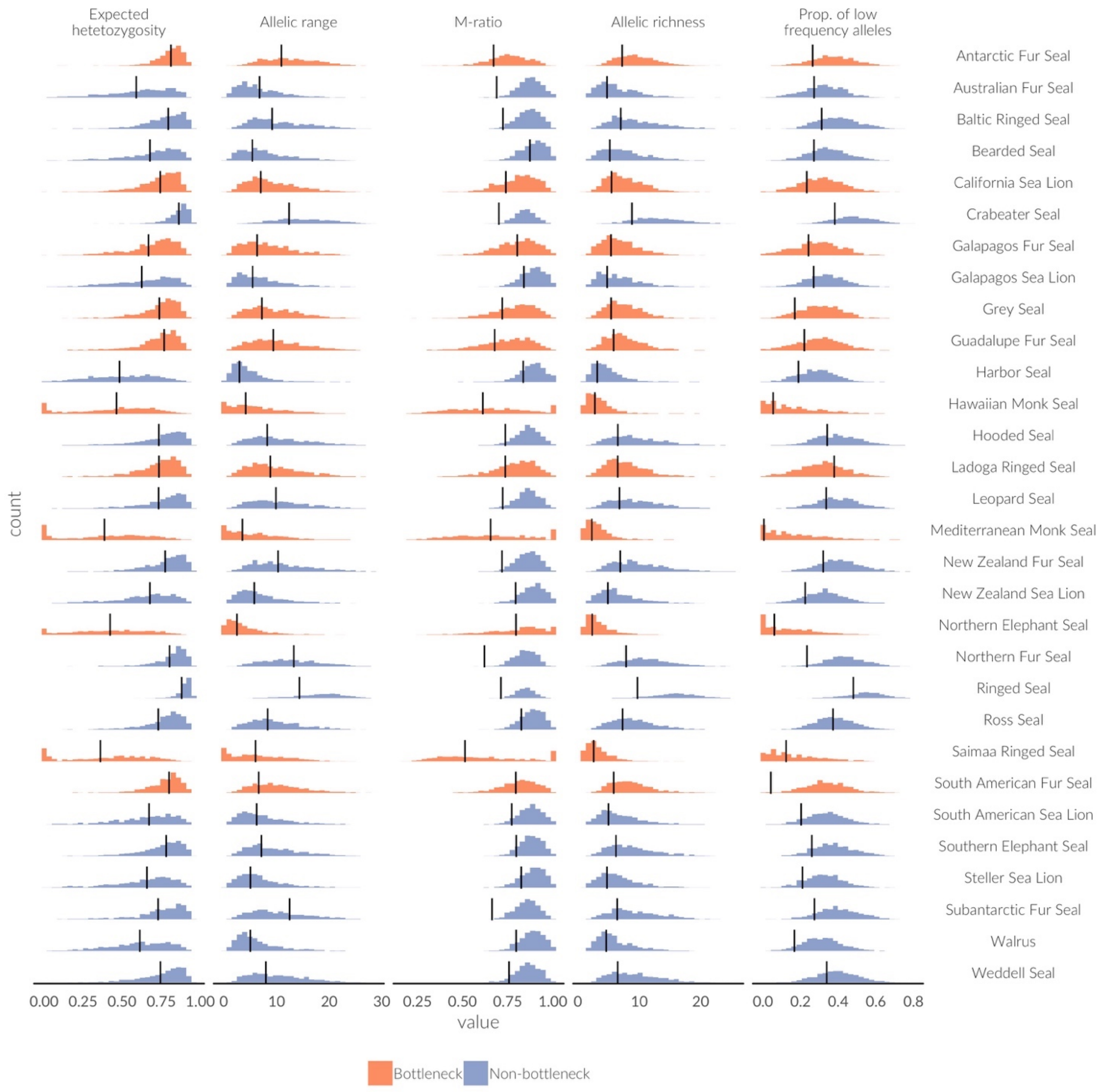

Supplementary Fig. 2: Posterior predictive checks for the summary statistics used in the ABC analysis (see Methods for details). After estimating posterior distributions of all parameters under the preferred model for each species (bottleneck versus non-bottleneck, colour coded orange and purple respectively), we sampled a set of 1,000 multivariate parameters from these distributions per species. Based on these parameters, we re-simulated data under the preferred model for each species to obtain 1,000 sets of summary statistics per species. The histograms show the distributions of these five summary statistics with the observed summary statistic of each species superimposed as a black vertical line. When interpreting these plots, it is important to bear in mind how informative a summary statistic can be for a given model. For example, the M-ratio is highly informative about recent bottlenecks and therefore showed good concordance between simulated and observed summary statistics for species supporting the bottleneck but not the non-bottleneck model.

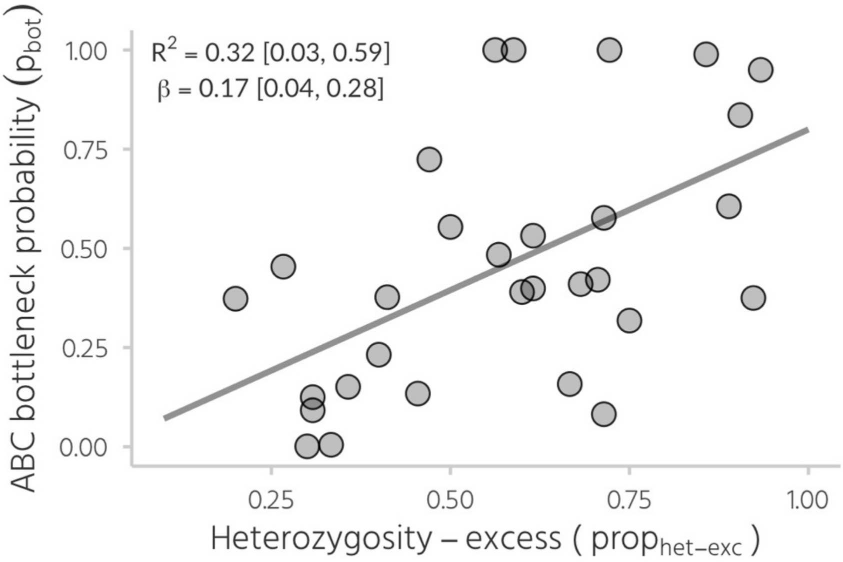

Supplementary Fig. 3: Correlation between the two bottleneck measures. Shown is a scatterplot of p_{bot} against $prop_{\text{het-exc}}$ with the regression line showing predicted values from a Bayesian phylogenetic mixed model (see Methods). Also shown are the marginal R^2 and the standardised β estimate of the model including 95% credible intervals.

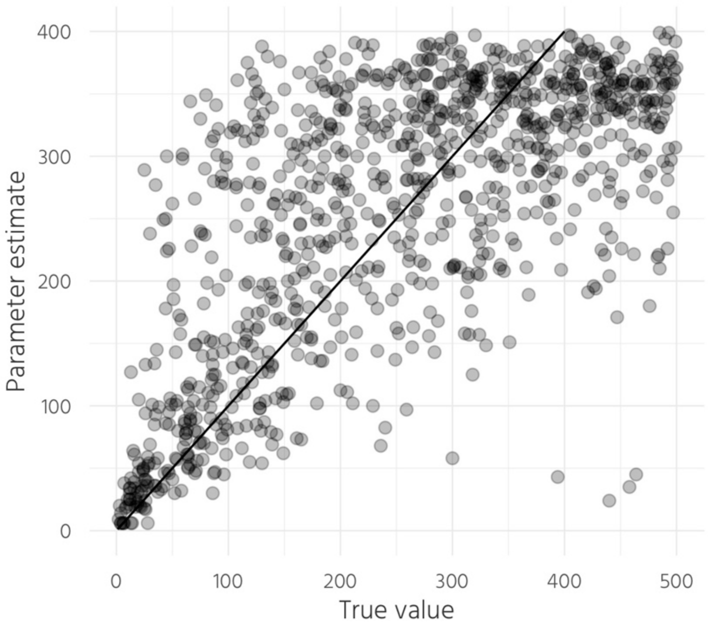

Supplementary Fig. 4: Scatter plot of the of the cross-validation evaluation of $N_e$bot. Shown are the true values plotted against the estimated values in our ABC analysis. The plot reflects a prediction error of 0.55 (see Supplementary Table 4).

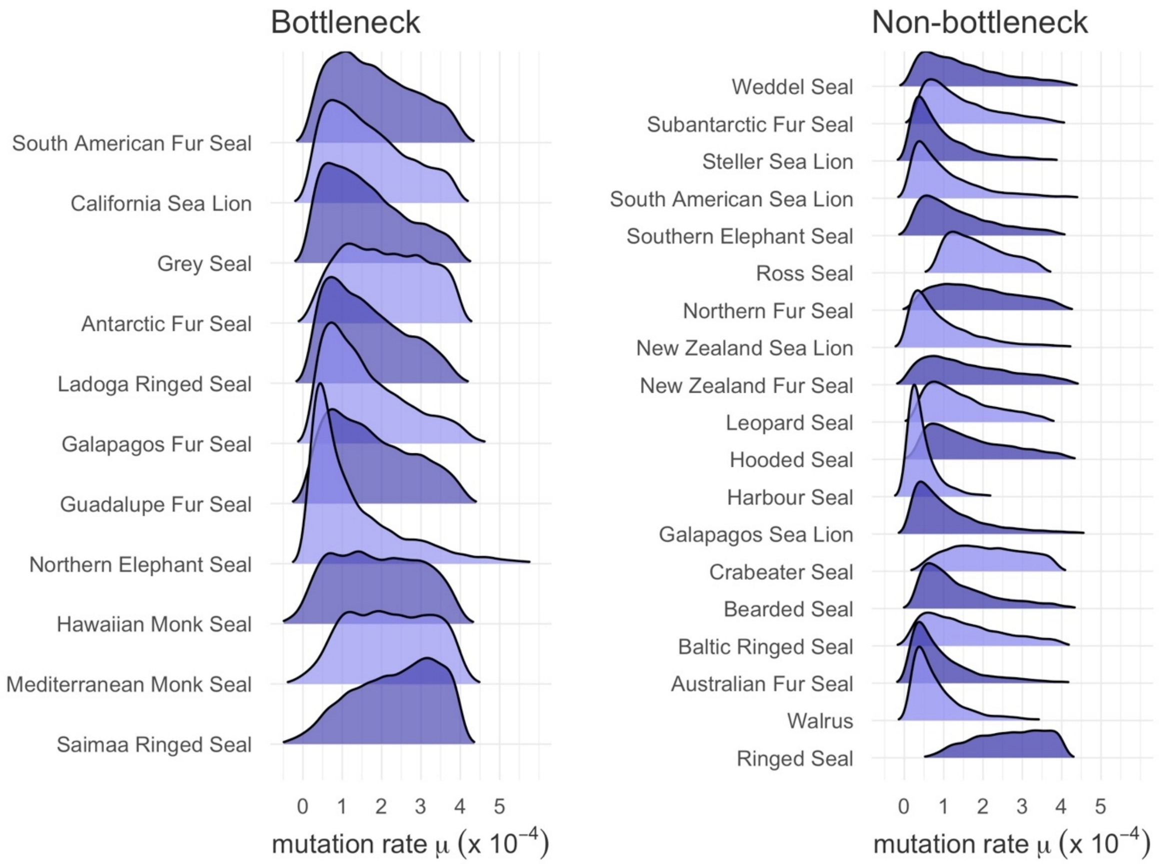

Supplementary Fig. 5: Ridgeline plots [1] of ABC posterior estimates of the microsatellite mutation rate (μ) for species supporting (A) the bottleneck model; and (B) the non-bottleneck model. μ was drawn from a uniform prior with $\mu \sim U[10^{-5}, 10^{-4}]$.

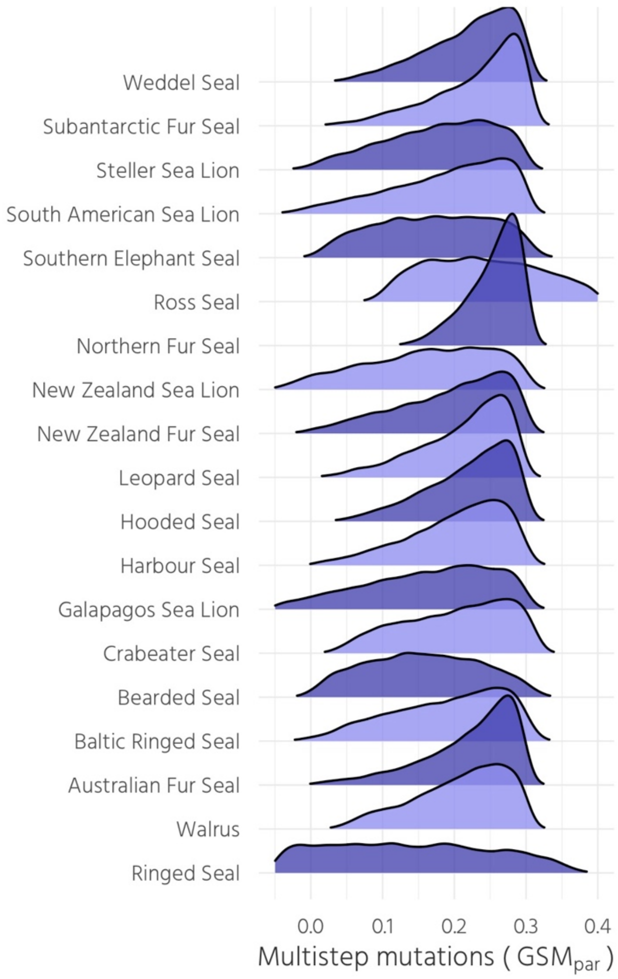

Supplementary Fig. 6: Ridgeline plots of ABC posterior estimates of the GSM parameter (*GSM_{par}*) for species supporting the non-bottleneck model. The parameter reflects the proportion of multistep mutations and was drawn from a uniform distribution with GSM_{par} ~U[0, 0.3].

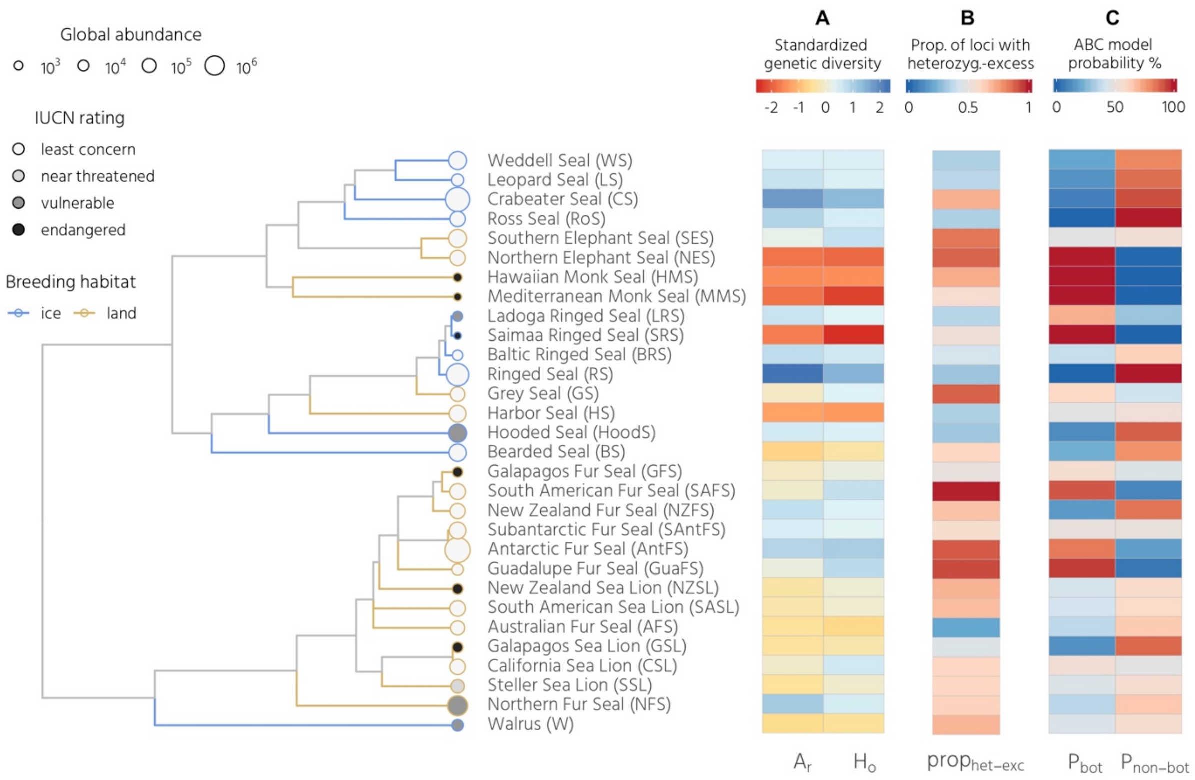

Supplementary Fig. 7: Replicated Figure 1 based on reduced datasets containing only individuals from the largest genetic cluster of each species.

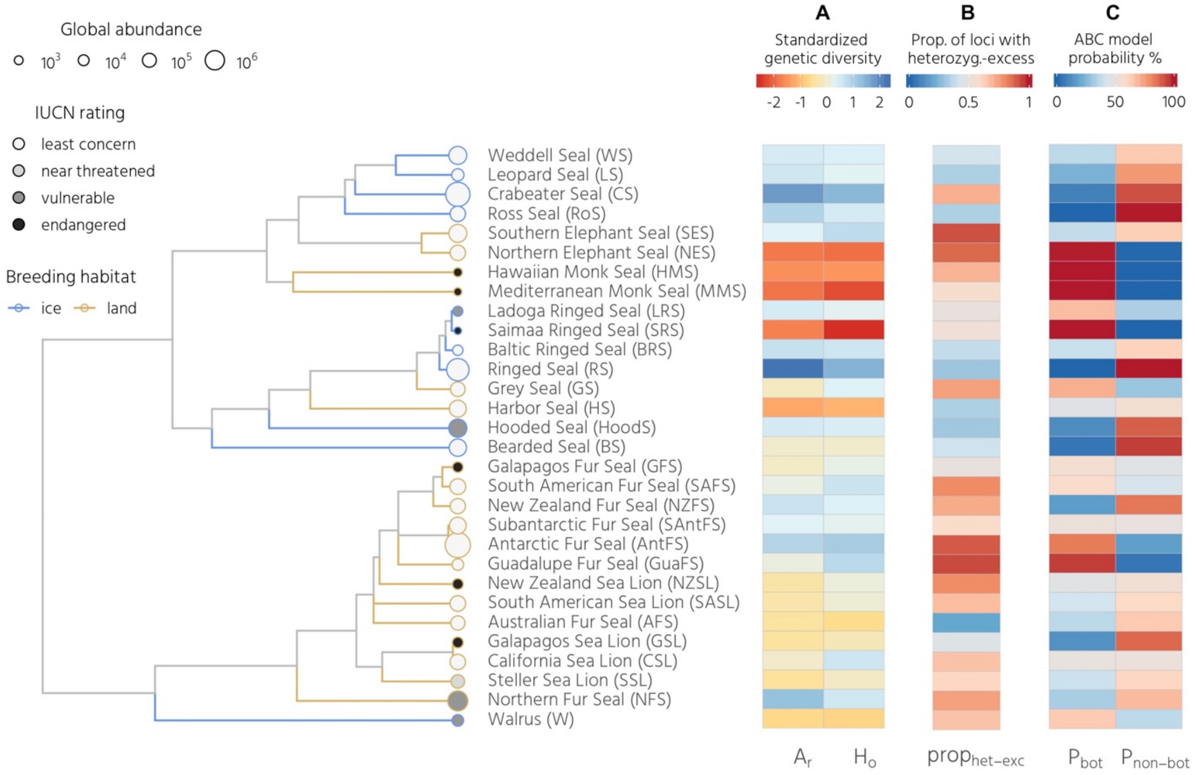

Supplementary Fig. 8: Replicated Figure 1 based on reduced datasets containing only loci in Hardy-Weinberg equilibrium.

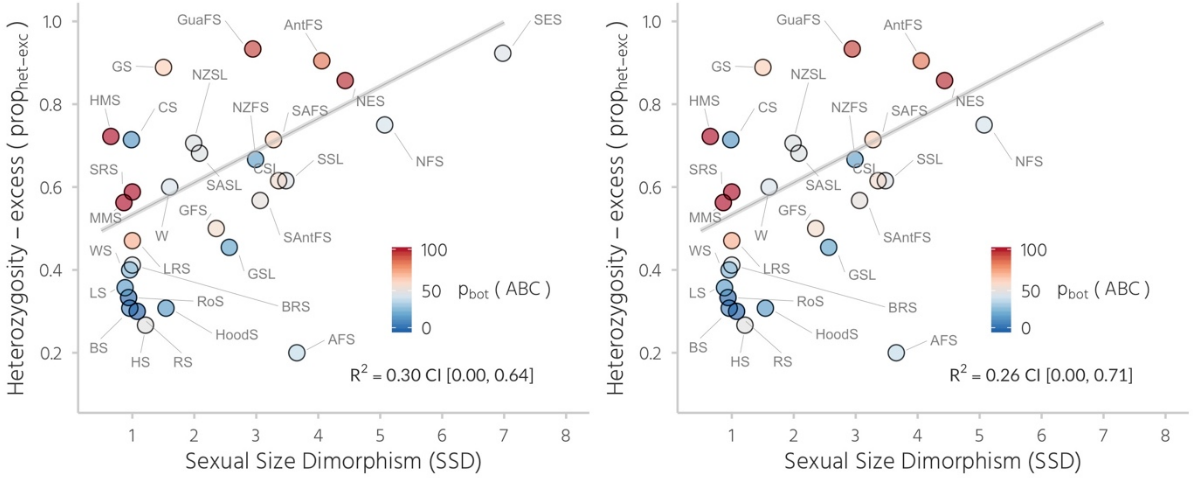

Supplementary Fig. 9: Robustness of the relationship between $prop_{het\text{-}exc}$ and SSD to the exclusion of the southern elephant seal (SES). The left panel shows the raw data and model prediction for the full dataset, while the right panel presents equivalent results for the dataset after excluding the southern elephant seal.

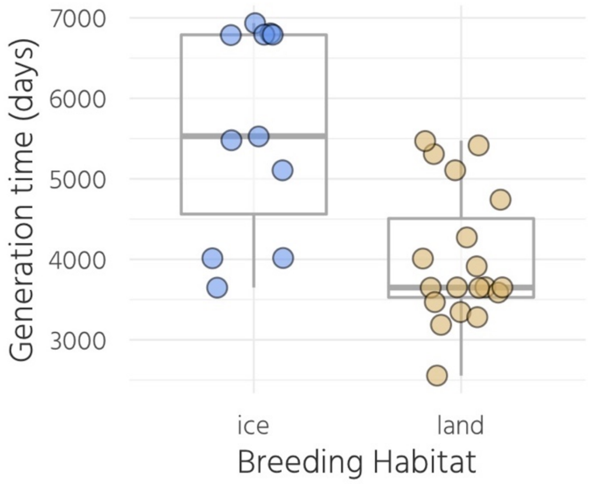

Supplementary Fig. 10: Differences in generation times by breeding habitat across all species.

		Conservation, demography, ecology and life-history data						*Genetic data*				
**Common name**	**Scientific name**	**IUCN status**	**Abundance**	**Breeding habitat**	**Generation time (days)**	**SSD**	**Breeding season length**	**Individuals**	**Loci**	**Genotypes**	**Sampling locations (n)**	**Publication**
Weddell Seal (WS)	*Leptonychotes weddellii*	least concern	633,000	ice	4015	0.957	45	893	15	13,395	Antarctica (7)	Davis et al. (2008)
Leopard Seal (LS)	*Hydrurga leptonyx*	least concern	35,500	ice	5110	0.883	100	150	14	2,100	Antarctica and sub-Antarctic Islands (6)	Davis et al. (2008)
Crabeater Seal (CS)	*Lobodon carcinophagus*	least concern	4,000,000	ice	5475	0.984	45	303	7	2,121	Antarctica (3)	Davis et al. (2008)
Ross Seal (RoS)	*Ommatophoca rossii*	least concern	220,000	ice	4015	0.939	60	90	9	810	Antarctica (4)	Davis et al. (2008)
Southern Elephant Seal (SES)	*Mirounga leonina*	least concern	650,000	land	3468	6.978	60	260	13	3,380	Falkland Islands (1)	This study
Northern Elephant Seal (NES)	*Mirounga angustirostris*	least concern	220,000	land	3184	4.435	70	260	35	9,100	San Benitos Archipelago, Mexico (3)	This study
Hawaiian Monk Seal (HMS)	*Monachus schauinslandi*	endangered	1,209	land	5475	0.653	120	2386	18	42,948	Hawaii (8)	Schultz et al. (2011)
Mediterranean Monk Seal (MMS)	*Monachus monachus*	endangered	400	land	4015	0.864	60	109	16	1,744	Western Sahara (1)	Pastor et al. (2004)
Ladoga Ringed Seal (LRS)	*Phoca hispida ladogensis*	vulnerable	4,000	ice	6789	1.000	60	16	17	272	Lake Ladoga, Russia (1)	Nyman et al. (2014)
Saimaa Ringed Seal (SRS)	*Phoca hispida saimensis*	endangered	280	ice	6789	1.000	60	172	17	2,924	Lake Saimaa, Finland (1)	Nyman et al. (2014)
Baltic Ringed Seal (BRS)	*Phoca hispida botnica*	least concern	6,500	ice	6789	1.000	60	21	17	357	Baltic Sea (1)	Nyman et al. (2014)
Ringed Seal (RS)	*Pusa hispida*	least concern	2,500,000	ice	6804	1.078	60	303	10	3,030	Arctic, several locations (8)	Davis et al. (2008)
Grey Seal (GS)	*Halichoerus grypus*	least concern	147,000	land	5110	1.503	60	1254	9	11,286	Orkney Archipelago, Scotland (13)	Klimova et al. (2014)
Harbor Seal (HS)	*Phoca vitulina*	least concern	425,000	land	5414	1.212	90	259	15	3,885	Waddensee, Denmark (1)	Rijks et al. (2017)
Hooded Seal (HoodS)	*Cystophora cristata*	vulnerable	662,000	ice	3650	1.542	20	300	13	3,900	Canada, Greenland Sea (4)	Coltman et al. (2007)
Bearded Seal (BS)	*Erignathus barbatus*	least concern	500,000	ice	6935	0.959	20	119	13	1,547	Arctic, several locations (6)	Davis et al. (2008)
Galapagos Fur Seal (GFS)	*Arctocephalus galapagoensis*	endangered	7,000	land	3650	2.354	90	90	12	1,080	Fernandina and Isabella Islands, Galapagos (3)	Lopes et al. (2015)
South American Fur Seal (SAFS)	*Arctocephalus australis*	least concern	275,000	land	4270	3.278	45	226	7	1,582	Brazil, Peru (2)	De Oliveira et al. (2008)
New Zealand Fur Seal (NZFS)	*Arctocephalus forsteri*	least concern	200,000	land	3285	2.989	45	54	12	648	Open Bay, New Zealand (1)	Dussex et al. (2016)
Subantarctic Fur Seal (SAntFS)	*Arctocephalus tropicalis*	least concern	400,000	land	3913	3.062	50	88	37	3,256	Macquarie Island (1)	This study
Antarctic Fur Seal (AntFS)	*Arctocephalus gazella*	least concern	5,000,000	land	3342	4.058	30	246	21	5,166	Bird Island, South Georgia (9)	Hoffman et al. (2011)
Guadalupe Fur Seal (GuaFS)	*Arctocephalus townsendi*	least concern	20,000	land	3650	2.944	45	224	15	3,360	Guadalupe Island (1)	This study
New Zealand Sea Lion (NZSL)	*Phocarctos hookeri*	endangered	9,880	land	3650	1.989	30	205	17	3,485	New Zealand (4)	Osborne et al. (2016)
South American Sea Lion (SASL)	*Otaria byronia / flavescens*	least concern	250,000	land	2555	2.083	180	270	22	5,940	Falkland Islands (23)	Hoffman et al. (2016)
Australian Fur Seal (AFS)	*Arctocephalus pusillus*	least concern	120,000	land	3588	3.655	60	183	5	915	Australia (9)	Lancaster et al. (2010)
Galapagos Sea Lion (GSL)	*Zalophus wollebaeki*	endangered	9,900	land	3650	2.564	180	781	22	17,182	Caamano, Galapagos (1)	This study
California Sea Lion (CSL)	*Zalophus californianus*	least concern	226,000	land	5307	3.360	60	347	13	4,511	San Miguel Island, California (1)	Acevedo-Whitehouse et al. (2006)
Steller Sea Lion (SSL)	*Eumetopias jubatus*	near threatened	78,000	land	3650	3.478	45	668	13	8,684	Aleutian Islands (32)	Hoffman et al. (2006)
Northern Fur Seal (NFS)	*Callorhinus ursinus*	vulnerable	1,290,000	land	4745	5.073	45	492	8	3,936	Aleutian Islands (8)	Dickerson et al. (2010)
Walrus (W)	*Odobenus rosmarus*	vulnerable	19,000	ice	5530	1.603	50	623	10	6,230	Atlantic population (6)	Shafer et al. (2014)

Supplementary Table 1: Summary of the pinniped species in this study including genetic, conservation and life history data. Abbreviations for the common names are given in parentheses. Demographic and life-history data for each species were obtained from [2] and data on conservation status were retrieved from the IUCN website (http://www.iucnredlist.org/, 2017)[3]. SSD (sexual size dimorphism) was calculated as the ratio of male to female body mass. Microsatellite data for five species were generated as part of this study, while the other data were originally generated in [4–18]. The species are presented in the same order as in the phylogeny in Fig. 1.

Common name	Scientific name	Ar	Ho	He	Proportion of low frequency alleles	Allelic range	M-ratio
Weddell Seal (WS)	*Leptonychotes weddellii*	6.45 (5.87, 7.07)	0.72 (0.65, 0.79)	0.74 (0.71, 0.78)	0.34 (0.26, 0.42)	7.96 (6.9, 9.23)	0.76 (0.69, 0.82)
Leopard Seal (LS)	*Hydrurga leptonyx*	6.73 (6.07, 7.36)	0.73 (0.67, 0.78)	0.73 (0.7, 0.77)	0.33 (0.24, 0.44)	9.85 (8.43, 11.31)	0.72 (0.64, 0.8)
Crabeater Seal (CS)	*Lobodon carcinophagus*	8.76 (7.85, 9.71)	0.85 (0.77, 0.93)	0.86 (0.83, 0.89)	0.38 (0.24, 0.5)	12.36 (10.43, 14)	0.7 (0.62, 0.78)
Ross Seal (RoS)	*Ommatophoca rossii*	7.23 (6.56, 8)	0.73 (0.63, 0.82)	0.73 (0.68, 0.77)	0.37 (0.23, 0.5)	8.3 (7.04, 9.5)	0.83 (0.73, 0.94)
Southern Elephant Seal (SES)	*Mirounga leonina*	6.15 (5.62, 6.69)	0.77 (0.69, 0.84)	0.78 (0.75, 0.81)	0.26 (0.16, 0.35)	7.11 (6.27, 7.96)	0.8 (0.73, 0.86)
Northern Elephant Seal (NES)	*Mirounga angustirostris*	2.28 (2.17, 2.37)	0.4 (0.35, 0.45)	0.42 (0.39, 0.45)	0.06 (0.02, 0.11)	2.48 (2.29, 2.64)	0.8 (0.77, 0.82)
Hawaiian Monk Seal (HMS)	*Monachus schauinslandi*	2.73 (2.5, 2.94)	0.46 (0.38, 0.53)	0.46 (0.42, 0.51)	0.05 (0, 0.13)	4.13 (3.67, 4.56)	0.61 (0.59, 0.64)
Mediterranean Monk Seal (MMS)	*Monachus monachus*	2.22 (1.94, 2.44)	0.34 (0.25, 0.43)	0.39 (0.32, 0.45)	0 (0, 0.04)	3.51 (2.79, 4.29)	0.65 (0.58, 0.73)
Ladoga Ringed Seal (LRS)	*Phoca hispida ladogensis*	6.45 (6.06, 6.88)	0.69 (0.65, 0.73)	0.74 (0.71, 0.76)	0.38 (0.31, 0.45)	8.8 (8.38, 9.18)	0.74 (0.7, 0.78)
Saimaa Ringed Seal (SRS)	*Phoca hispida saimensis*	2.54 (2.24, 2.76)	0.33 (0.27, 0.4)	0.36 (0.31, 0.4)	0.12 (0.04, 0.22)	6.02 (5.17, 6.79)	0.51 (0.47, 0.56)
Baltic Ringed Seal (BRS)	*Phoca hispida botnica*	6.94 (6.41, 7.53)	0.74 (0.69, 0.79)	0.79 (0.77, 0.82)	0.31 (0.25, 0.38)	9.14 (8.53, 9.76)	0.72 (0.68, 0.76)
Ringed Seal (RS)	*Pusa hispida*	9.64 (8.7, 10.6)	0.87 (0.8, 0.93)	0.88 (0.85, 0.91)	0.48 (0.39, 0.57)	14.3 (11.5, 21.35)	0.71 (0.62, 0.8)
Grey Seal (GS)	*Halichoerus grypus*	5.36 (4.78, 6)	0.72 (0.63, 0.8)	0.74 (0.69, 0.78)	0.17 (0.04, 0.32)	7.21 (6, 8)	0.72 (0.64, 0.8)
Harbor Seal (HS)	*Phoca vitulina*	3.1 (2.8, 3.4)	0.47 (0.39, 0.53)	0.48 (0.44, 0.53)	0.19 (0.1, 0.28)	2.95 (2.47, 3.47)	0.84 (0.77, 0.89)
Hooded Seal (HoodS)	*Cystophora cristata*	6.47 (5.77, 7.16)	0.72 (0.65, 0.79)	0.73 (0.69, 0.77)	0.34 (0.23, 0.44)	8.21 (7.15, 9.31)	0.74 (0.67, 0.8)
Bearded Seal (BS)	*Erignathus barbatus*	5.14 (4.46, 5.77)	0.62 (0.55, 0.69)	0.68 (0.63, 0.72)	0.27 (0.18, 0.37)	5.39 (4.38, 6.31)	0.87 (0.81, 0.93)
Galapagos Fur Seal (GFS)	*Arctocephalus galapagoensis*	5.34 (4.83, 5.83)	0.68 (0.61, 0.75)	0.67 (0.62, 0.71)	0.24 (0.13, 0.35)	6.31 (5.12, 7.5)	0.8 (0.72, 0.88)
South American Fur Seal (SAFS)	*Arctocephalus australis*	5.77 (5, 6.57)	0.77 (0.65, 0.86)	0.8 (0.74, 0.84)	0.04 (0, 0.16)	6.61 (5.57, 9)	0.79 (0.67, 0.88)
New Zealand Fur Seal (NZFS)	*Arctocephalus forsteri*	6.86 (6.25, 7.5)	0.72 (0.65, 0.79)	0.77 (0.74, 0.81)	0.32 (0.22, 0.41)	10.28 (9.25, 11.33)	0.72 (0.67, 0.77)
Subantarctic Fur Seal (SAntFS)	*Arctocephalus tropicalis*	6.36 (5.89, 6.89)	0.71 (0.67, 0.75)	0.73 (0.71, 0.75)	0.27 (0.19, 0.34)	12.46 (11.16, 13.64)	0.66 (0.62, 0.7)
Antarctic Fur Seal (AntFS)	*Arctocephalus gazella*	7.16 (6.67, 7.62)	0.81 (0.75, 0.86)	0.81 (0.79, 0.83)	0.26 (0.03, 0.36)	10.91 (9.86, 11.95)	0.67 (0.61, 0.73)
Guadalupe Fur Seal (GuaFS)	*Arctocephalus townsendi*	5.76 (5.33, 6.2)	0.77 (0.71, 0.84)	0.77 (0.74, 0.79)	0.22 (0.15, 0.3)	9.36 (8.73, 9.93)	0.68 (0.64, 0.71)
New Zealand Sea Lion (NZSL)	*Phocarctos hookeri*	4.84 (4.41, 5.24)	0.67 (0.6, 0.74)	0.68 (0.64, 0.71)	0.22 (0.11, 0.31)	5.78 (5.03, 6.41)	0.79 (0.74, 0.85)
South American Sea Lion (SASL)	*Otaria byronia / flavescens*	4.93 (4.57, 5.33)	0.66 (0.6, 0.72)	0.67 (0.64, 0.7)	0.2 (0.11, 0.3)	6.22 (5.59, 6.77)	0.77 (0.73, 0.81)
Australian Fur Seal (AFS)	*Arctocephalus pusillus*	4.7 (4, 5.4)	0.58 (0.46, 0.7)	0.59 (0.5, 0.66)	0.27 (0.12, 0.44)	6.76 (5.2, 8.5)	0.69 (0.56, 0.81)
Galapagos Sea Lion (GSL)	*Zalophus wollebaeki*	4.72 (4.32, 5.09)	0.62 (0.57, 0.68)	0.62 (0.59, 0.66)	0.27 (0.2, 0.34)	5.45 (4.86, 6.32)	0.84 (0.79, 0.88)
California Sea Lion (CSL)	*Zalophus californianus*	5.42 (4.85, 6)	0.74 (0.67, 0.81)	0.74 (0.71, 0.78)	0.23 (0.13, 0.33)	6.99 (6.15, 7.92)	0.74 (0.67, 0.8)
Steller Sea Lion (SSL)	*Eumetopias jubatus*	4.69 (4.23, 5.15)	0.64 (0.56, 0.72)	0.66 (0.61, 0.7)	0.21 (0.12, 0.31)	5.02 (4.31, 5.85)	0.82 (0.76, 0.88)
Northern Fur Seal (NFS)	*Callorhinus ursinus*	7.81 (6.88, 8.75)	0.74 (0.66, 0.82)	0.8 (0.76, 0.85)	0.23 (0, 0.46)	13.25 (10.25, 15.14)	0.62 (0.54, 0.72)
Walrus (W)	*Odobenus rosmarus*	4.56 (4, 5.1)	0.58 (0.49, 0.68)	0.61 (0.55, 0.67)	0.17 (0.06, 0.29)	5 (4.2, 5.8)	0.8 (0.72, 0.88)

Supplementary Table 2: Genetic summary statistics for 30 pinniped species. Abbreviations for the common names are given in parentheses. All statistics were calculated as the mean and 95% confidence interval across 1000 subsamples of 10 individuals each. The proportion of low frequency alleles was calculated as the number of alleles with frequencies below 5% and the allelic range is given as the number of repeat units between the smallest and largest allele. A_r allelic richness; H_o observed heterozygosity; H_e expected heterozygosity.

		Heterozygosity-excess ($prop_{het-exc}$)				ABC	
**Common name**	**Scientific name**	**TPM 70**	**TPM 80**	**TPM 90**	**SMM**	p_{bot}	$p_{non-bot}$
Weddell Seal (WS)	*Leptonychotes weddellii*	0.467	0.400	0.267	0.067	0.232	0.768
Leopard Seal (LS)	*Hydrurga leptonyx*	0.357	0.357	0.286	0.143	0.151	0.849
Crabeater Seal (CS)	*Lobodon carcinophagus*	0.714	0.714	0.571	0.143	0.082	0.918
Ross Seal (RoS)	*Ommatophoca rossii*	0.333	0.333	0.222	0.111	0.005	0.995
Southern Elephant Seal (SES)	*Mirounga leonina*	0.923	0.923	0.846	0.385	0.375	0.625
Northern Elephant Seal (NES)	*Mirounga angustirostris*	0.857	0.857	0.857	0.829	0.989	0.011
Hawaiian Monk Seal (HMS)	*Monachus schauinslandi*	0.833	0.722	0.722	0.444	1.000	0.000
Mediterranean Monk Seal (MMS)	*Monachus monachus*	0.562	0.562	0.562	0.562	1.000	0.000
Ladoga Ringed Seal (LRS)	*Phoca hispida ladogensis*	0.471	0.471	0.353	0.176	0.724	0.276
Saimaa Ringed Seal (SRS)	*Phoca hispida saimensis*	0.588	0.588	0.529	0.529	1.000	0.000
Baltic Ringed Seal (BRS)	*Phoca hispida botnica*	0.412	0.412	0.353	0.235	0.377	0.623
Ringed Seal (RS)	*Pusa hispida*	0.400	0.300	0.200	0.000	0.001	0.999
Grey Seal (GS)	*Halichoerus grypus*	0.889	0.889	0.778	0.444	0.606	0.394
Harbor Seal (HS)	*Phoca vitulina*	0.533	0.267	0.133	0.067	0.454	0.546
Hooded Seal (HoodS)	*Cystophora cristata*	0.385	0.308	0.308	0.077	0.125	0.875
Bearded Seal (BS)	*Erignathus barbatus*	0.308	0.308	0.308	0.231	0.092	0.908
Galapagos Fur Seal (GFS)	*Arctocephalus galapagoensis*	0.500	0.500	0.500	0.417	0.554	0.446
South American Fur Seal (SAFS)	*Arctocephalus australis*	0.857	0.714	0.571	0.143	0.577	0.423
New Zealand Fur Seal (NZFS)	*Arctocephalus forsteri*	0.667	0.667	0.583	0.417	0.158	0.842
Subantarctic Fur Seal (SAntFS)	*Arctocephalus tropicalis*	0.622	0.568	0.432	0.189	0.484	0.516
Antarctic Fur Seal (AntFS)	*Arctocephalus gazella*	0.952	0.905	0.905	0.571	0.836	0.164
Guadalupe Fur Seal (GuaFS)	*Arctocephalus townsendi*	1.000	0.933	0.933	0.600	0.950	0.050
New Zealand Sea Lion (NZSL)	*Phocarctos hookeri*	0.706	0.706	0.471	0.353	0.421	0.579
South American Sea Lion (SASL)	*Otaria byronia / flavescens*	0.773	0.682	0.455	0.182	0.410	0.590
Australian Fur Seal (AFS)	*Arctocephalus pusillus*	0.400	0.200	0.200	0.000	0.373	0.627
Galapagos Sea Lion (GSL)	*Zalophus wollebaeki*	0.545	0.455	0.273	0.091	0.134	0.866
California Sea Lion (CSL)	*Zalophus californianus*	0.692	0.615	0.538	0.231	0.532	0.468
Steller Sea Lion (SSL)	*Eumetopias jubatus*	0.692	0.615	0.538	0.308	0.399	0.601
Northern Fur Seal (NFS)	*Callorhinus ursinus*	0.750	0.750	0.500	0.125	0.318	0.682
Walrus (W)	*Odobenus rosmarus*	0.700	0.600	0.500	0.100	0.390	0.610

Supplementary Table 3: Summary of bottleneck signatures across 30 pinniped species. Shown is the proportion of loci in heterozygosity-excess ($prop_{\text{het-exc}}$) corresponding to four mutation models with a decreasing proportion of multi-step mutations as well as the model selection results of the ABC analysis showing posterior probabilities for the bottleneck(p_{bot}) and non-bottleneck model ($p_{\text{non-bot}}$) respectively. See Methods for details.

A	Bottleneck model									
	N_ebot (E_{pred}= 0.54)					μ (E_{pred}= 0.75)				
Common name	mean	median	mode	HPD lower	HPD upper	mean	median	mode	HPD lower	HPD upper
Antarctic Fur Seal (AntFS)	320	327	370	127	497	2.07e-04	2.02e-04	1.46e-04	4.62e-05	3.91e-04
California Sea Lion (CSL)	323	330	394	116	505	1.64e-04	1.46e-04	9.84e-05	1.59e-05	3.57e-04
Galapagos Fur Seal (GFS)	300	303	299	128	459	1.60e-04	1.34e-04	9.22e-05	2.14e-05	3.71e-04
Grey Seal (GS)	310	314	316	110	501	1.63e-04	1.45e-04	9.70e-05	1.61e-05	3.59e-04
Guadalupe Fur Seal (GuaFS)	263	254	220	61	485	1.75e-04	1.58e-04	1.03e-04	1.69e-05	3.79e-04
Hawaiian Monk Seal (HMS)	50	41	32	3	121	1.89e-04	1.85e-04	1.33e-04	1.32e-05	3.84e-04
Ladoga Ringed Seal (LRS)	306	309	319	122	491	1.63e-04	1.46e-04	9.44e-05	1.78e-05	3.46e-04
Mediterranean Monk Seal (MMS)	36	29	24	3	83	2.20e-04	2.18e-04	1.89e-04	3.84e-05	3.99e-04
Northern Elephant Seal (NES)	69	47	33	2	203	1.33e-04	8.83e-05	6.03e-05	7.78e-06	3.88e-04
Saimaa Ringed Seal (SRS)	35	30	25	5	75	2.34e-04	2.47e-04	3.04e-04	4.48e-05	4.02e-04
South American Fur Seal (SAFS)	332	348	403	124	500	1.81e-04	1.68e-04	1.17e-04	1.99e-05	3.70e-04

B	Non-bottleneck model									
	GSM_{par} (E_{pred}= 0.84)					μ (E_{pred}= 0.70)				
Common name	mean	median	mode	HPD lower	HPD upper	mean	median	mode	HPD lower	HPD upper
Ringed Seal (RS)	0.133	0.127	0.086	-0.05	0.34	2.66e-04	2.73e-04	3.24e-04	1.13e-04	4.05e-04
Walrus (W)	0.208	0.22	0.247	0.082	0.301	8.57e-05	6.26e-05	4.40e-05	1.13e-05	2.42e-04
Australian Fur Seal (AFS)	0.221	0.239	0.262	0.088	0.301	1.02e-04	7.09e-05	4.90e-05	3.47e-06	3.01e-04
Baltic Ringed Seal (BRS)	0.189	0.201	0.243	0.041	0.303	1.52e-04	1.30e-04	8.68e-05	7.95e-06	3.59e-04
Bearded Seal (BS)	0.156	0.156	0.152	0.016	0.293	1.41e-04	1.12e-04	8.20e-05	2.16e-05	3.44e-04
Crabeater Seal (CS)	0.202	0.212	0.253	0.066	0.311	2.12e-04	2.07e-04	1.69e-04	5.69e-05	3.77e-04
Galapagos Sea Lion (GSL)	0.163	0.175	0.215	-0.005	0.303	1.17e-04	8.60e-05	5.85e-05	8.45e-06	3.30e-04
Harbor Seal (HS)	0.203	0.215	0.242	0.073	0.306	4.69e-05	3.31e-05	2.42e-05	8.39e-06	1.31e-04
Hooded Seal (HoodS)	0.22	0.233	0.258	0.104	0.304	1.66e-04	1.42e-04	9.77e-05	2.52e-05	3.66e-04
Leopard Seal (LS)	0.213	0.229	0.252	0.091	0.298	1.46e-04	1.26e-04	9.20e-05	2.84e-05	3.19e-04
New Zealand Fur Seal (NZFS)	0.195	0.211	0.246	0.049	0.302	1.66e-04	1.43e-04	9.20e-05	1.89e-06	3.74e-04
New Zealand Sea Lion (NZSL)	0.163	0.172	0.21	0.004	0.305	1.01e-04	7.23e-05	4.71e-05	2.13e-06	2.94e-04
Northern Fur Seal (NFS)	0.255	0.264	0.278	0.182	0.302	1.86e-04	1.72e-04	1.29e-04	2.55e-05	3.74e-04
Ross Seal (RoS)	0.247	0.241	0.219	0.102	0.401	1.87e-04	1.75e-04	1.40e-04	8.25e-05	3.30e-04
Southern Elephant Seal (SES)	0.171	0.173	0.194	0.033	0.303	1.37e-04	1.13e-04	7.77e-05	1.12e-05	3.37e-04
South American Sea Lion (SASL)	0.189	0.205	0.242	0.034	0.305	1.08e-04	7.79e-05	5.30e-05	7.95e-06	3.12e-04
Steller Sea Lion (SSL)	0.179	0.187	0.22	0.037	0.3	9.42e-05	6.78e-05	4.64e-05	6.71e-06	2.70e-04
Subantarctic Fur Seal (SAntFS)	0.23	0.247	0.271	0.104	0.308	1.42e-04	1.17e-04	8.45e-05	2.52e-05	3.26e-04
Weddell Seal (WS)	0.221	0.233	0.259	0.104	0.307	1.52e-04	1.27e-04	8.54e-05	1.42e-05	3.65e-04

Supplementary Table 4: Summary of ABC posterior estimates under (A) the bottleneck model; and (B) the non-bottleneck model. For the eleven species for which the bottleneck model was supported in the ABC analysis, summary statistics of the posterior distributions are given for two estimated model parameters: the bottleneck effective population size (N_ebot) and mutation rate (μ). For the 19 species for which the non-bottleneck model was supported in the ABC analysis, summary statistics of the posterior distributions are given for two estimated model parameters: the proportion of multi-step mutations (GSM_{par}) the mutation rate (μ). The mean and medians of all summary statistics are based on 5000 accepted parameter values for each species, while the modal values represent the respective modes of their density curves as depicted in Fig. 2. Also shown are the 95% highest posterior density intervals as calculated with the HPDinterval function in MCMCglmm. The prediction error from the leave-one-out cross-validation for each parameter is denoted E_{pred} and ranges from 0 to 1, whereby values smaller than 1 indicate that the posterior estimate contains information about the true underlying parameter value. E_{pred} is calculated as $E_{pred} = \frac{\sum_i(\tilde{\theta}_i - \theta_i)^2}{Var(\theta_i)}$ where θ_i is the true parameter value of the ith simulated data set and $\tilde{\theta}_i$ is the ABC estimated parameter value (the posterior median)[19].

Common name	Scientific name	Selected model	*p* value
Weddell Seal (WS)	*Leptonychotes weddellii*	non-bottleneck	0.95
Leopard Seal (LS)	*Hydrurga leptonyx*	non-bottleneck	0.91
Crabeater Seal (CS)	*Lobodon carcinophagus*	non-bottleneck	0.69
Ross Seal (RoS)	*Ommatophoca rossii*	non-bottleneck	0.57
Southern Elephant Seal (SES)	*Mirounga leonina*	non-bottleneck	0.83
Northern Elephant Seal (NES)	*Mirounga angustirostris*	bottleneck	0.15
Hawaiian Monk Seal (HMS)	*Monachus schauinslandi*	bottleneck	0.21
Mediterranean Monk Seal (MMS)	*Monachus monachus*	bottleneck	0.21
Ladoga Ringed Seal (LRS)	*Phoca hispida ladogensis*	bottleneck	0.95
Saimaa Ringed Seal (SRS)	*Phoca hispida saimensis*	bottleneck	0.14
Baltic Ringed Seal (BRS)	*Phoca hispida botnica*	non-bottleneck	0.79
Ringed Seal (RS)	*Pusa hispida*	non-bottleneck	0.31
Grey Seal (GS)	*Halichoerus grypus*	bottleneck	0.93
Harbor Seal (HS)	*Phoca vitulina*	non-bottleneck	0.96
Hooded Seal (HoodS)	*Cystophora cristata*	non-bottleneck	0.98
Bearded Seal (BS)	*Erignathus barbatus*	non-bottleneck	0.71
Galapagos Fur Seal (GFS)	*Arctocephalus galapagoensis*	bottleneck	0.92
South American Fur Seal (SAFS)	*Arctocephalus australis*	bottleneck	0.71
New Zealand Fur Seal (NZFS)	*Arctocephalus forsteri*	non-bottleneck	0.82
Subantarctic Fur Seal (SAntFS)	*Arctocephalus tropicalis*	non-bottleneck	0.99
Antarctic Fur Seal (AntFS)	*Arctocephalus gazella*	bottleneck	0.78
Guadalupe Fur Seal (GuaFS)	*Arctocephalus townsendi*	bottleneck	0.91
New Zealand Sea Lion (NZSL)	*Phocarctos hookeri*	non-bottleneck	0.99
South American Sea Lion (SASL)	*Otaria byronia / flavescens*	non-bottleneck	0.98
Australian Fur Seal (AFS)	*Arctocephalus pusillus*	non-bottleneck	0.99
Galapagos Sea Lion (GSL)	*Zalophus wollebaeki*	non-bottleneck	0.91
California Sea Lion (CSL)	*Zalophus californianus*	bottleneck	0.93
Steller Sea Lion (SSL)	*Eumetopias jubatus*	non-bottleneck	0.98
Northern Fur Seal (NFS)	*Callorhinus ursinus*	non-bottleneck	0.77
Walrus (W)	*Odobenus rosmarus*	non-bottleneck	0.88

Supplementary Table 5: Goodness of fit test for selected models across species. Shown is the selected model for each species and the corresponding p-value from 100 leave-one-out cross-validation replicates. A non-significant p-value indicates that the distance between the observed summary statistics and the accepted summary statistics from the ABC analysis is not larger than the expectation based on pseudo-observed data sets, i.e. the assigned model provides a good fit to the observed data.

Common name	Scientific name	Number of genetic clusters	Number of individuals in largest cluster
Weddell Seal (WS)	*Leptonychotes weddellii*	3	453
Leopard Seal (LS)	*Hydrurga leptonyx*	1	150
Crabeater Seal (CS)	*Lobodon carcinophagus*	1	303
Ross Seal (RoS)	*Ommatophoca rossii*	1	90
Southern Elephant Seal (SES)	*Mirounga leonina*	4	87
Northern Elephant Seal (NES)	*Mirounga angustirostris*	2	136
Hawaiian Monk Seal (HMS)	*Monachus schauinslandi*	1	2386
Mediterranean Monk Seal (MMS)	*Monachus monachus*	1	109
Ladoga Ringed Seal (LRS)	*Phoca hispida ladogensis*	2	13
Saimaa Ringed Seal (SRS)	*Phoca hispida saimensis*	2	148
Baltic Ringed Seal (BRS)	*Phoca hispida botnica*	1	21
Ringed Seal (RS)	*Pusa hispida*	1	303
Grey Seal (GS)	*Halichoerus grypus*	1	1254
Harbor Seal (HS)	*Phoca vitulina*	2	161
Hooded Seal (HoodS)	*Cystophora cristata*	1	300
Bearded Seal (BS)	*Erignathus barbatus*	2	65
Galapagos Fur Seal (GFS)	*Arctocephalus galapagoensis*	1	90
South American Fur Seal (SAFS)	*Arctocephalus australis*	2	142
New Zealand Fur Seal (NZFS)	*Arctocephalus forsteri*	1	54
Subantarctic Fur Seal (SAntFS)	*Arctocephalus tropicalis*	2	63
Antarctic Fur Seal (AntFS)	*Arctocephalus gazella*	1	246
Guadalupe Fur Seal (GuaFS)	*Arctocephalus townsendi*	1	224
New Zealand Sea Lion (NZSL)	*Phocarctos hookeri*	1	205
South American Sea Lion (SASL)	*Otaria byronia / Otaria flavescens*	1	270
Australian Fur Seal (AFS)	*Arctocephalus pusillus*	1	183
Galapagos Sea Lion (GSL)	*Zalophus wollebaeki*	1	781
California Sea Lion (CSL)	*Zalophus californianus*	1	347
Steller Sea Lion (SSL)	*Eumetopias jubatus*	2	374
Northern Fur Seal (NFS)	*Callorhinus ursinus*	2	249
Walrus (W)	*Odobenus rosmarus*	2	433

Supplementary Table 6: Summary of the STRUCTURE results. Shown is the most likely number of genetic clusters (see Methods for details) together with the number of individuals in the largest cluster.

variable	R	CI
A_r	0.994	[0.986 0.996]
H_o	0.999	[0.997 0.999]
TPM 70	0.934	[0.902 0.967]
TPM 80	0.916	[0.884 0.941]
TPM 90	0.968	[0.923 0.982]
SMM	0.972	[0.953 0.982]
p_{bot}	0.979	[0.952 0.987]

Supplementary Table 7: Consistency of results obtained using the full datasets and the largest genetic clusters. Repeatabilities (R) and 95 % confidence intervals (CI) are shown for the genetic summary statistics (A_r Allelic richness; H_o observed heterozygosity), heterozygosity-excess across four different mutation models (TPM 70–SMM) and the ABC bottleneck probability (p_{bot}).

variable	R	CI
A_r	0.998	[0.998 0.999]
H_o	0.996	[0.989 0.998]
TPM 70	0.983	[0.976 0.994]
TPM 80	0.975	[0.95 0.982]
TPM 90	0.988	[0.974 0.994]
SMM	0.988	[0.982 0.992]
p_{bot}	0.982	[0.97 0.992]

Supplementary Table 8: Consistency of results obtained using the full datasets and datasets with loci deviating from HWE removed. Repeatabilities (R) and 95 % confidence intervals (CI) are shown for genetic summary statistics (A_r Allelic richness; H_o observed heterozygosity), heterozygosity-excess across four different mutation models (TPM 70–SMM) and the ABC bottleneck probability (p_{bot}).

	Abundance	SSD	$prop_{het-exc}$	p_{bot}	Generation time	Breeding season length	Breeding Habitat
Abundance		0.13	0.002	0.280	0.040	0.076	0.000
SSD			0.277	0.013	0.285	0.010	0.368
$prop_{het-exc}$				0.280	0.115	0.004	0.265
p_{bot}					0.003	0.001	0.174
Generation time						0.031	0.389
Breeding season length							0.061
Breeding Habitat							

Supplementary Table 9: Correlations among predictor variables of genetic diversity (corresponding to Figure 3). Shown are the coefficients of determination (R^2) from linear models between all pairs of predictor variables used in the phylogenetic mixed model of allelic richness.

Model	stand. β	R^2_{unique}	$r(\hat{Y},x)$	$R^2_{marginal}$	$R^2_{conditional}$
**1**					
$\mathbf{prop}_{het-exc}$ ~					
SSD +	0.03 (-0.04, 0.11)	0.02 (-0.48, 0.57)	0.76 (0.35, 0.98)	0.58 (0.22, 0.92)	0.62 (0.27, 0.99)
Breeding Habitat +	-0.25 (-0.54, 0)	0.18 (-0.39, 0.67)	-0.85 (-1, -0.5)		
Generation time +	-0.04 (-0.16, 0.06)	0.01 (-0.38, 0.41)	-0.58 (-0.92, -0.03)		
Breeding season length	-0.52 (-0.92, -0.14)	0.25 (-0.14, 0.55)	-0.09 (-0.56, 0.33)		
**2**					
$\mathbf{p}_{bot}$ ~					
SSD +	0.03 (-0.04, 0.11)	0.02 (-0.48, 0.57)	0.28 (-0.42, 0.81)	0.38 (0.08, 0.62)	0.4 (0.13, 0.68)
Breeding Habitat +	-0.25 (-0.54, 0)	0.18 (-0.39, 0.67)	-0.79 (-1, -0.2)		
Generation time +	-0.04 (-0.16, 0.06)	0.01 (-0.38, 0.41)	-0.22 (-0.84, 0.45)		
Breeding season length	-0.52 (-0.92, -0.14)	0.25 (-0.14, 0.55)	0.04 (-0.56, 0.55)		
**3**					
$\mathbf{A}_r$ ~					
SSD +	1.38 (0.21, 2.47)	0 (-0.3, 0.33)	0.01 (-0.26, 0.36)	0.75 (0.52, 0.91)	0.76 (0.53, 0.93)
Breeding Habitat +	0.22 (-1.17, 1.58)	0.1 (-0.21, 0.44)	0.54 (0.2, 0.76)		
Abundance +	1.76 (0.1, 3.14)	0.1 (-0.22, 0.44)	0.73 (0.54, 0.91)		
p_{bot} +	-1.8 (-3.08, -0.42)	0.12 (-0.2, 0.46)	-0.78 (-0.91, -0.62)		
$prop_{het-exc}$	1.13 (-0.03, 2.42)	0 (-0.31, 0.36)	-0.16 (-0.42, 0.12)		
**4**					
$\mathbf{A}_r$ ~					
IUCN status	1.19 (-0.13, 2.64)			0.1 (0, 0.32)	0.11 (0, 0.34)
**5**					
$\mathbf{prop}_{het-exc}$ ~					
IUCN status	0.02 (-0.14, 0.19)			0.02 (0, 0.18)	0.09 (0.01, 0.36)
**6**					
$\mathbf{p}_{bot}$ ~					
IUCN status	-0.16 (-0.4, 0.1)			0.07 (0, 0.28)	0.1 (0, 0.32)

Supplementary Table 10: Estimated parameters for our Bayesian phylogenetic mixed models. Shown is the posterior median including 95% credible intervals (CI) summarizing the estimated statistics of the models from Figure 3 (models 1,2), Figure 4 (models 3), and Figure 5 (models 4,5 and 6). The response of each model is displayed at the beginning and followed by a ~. In subsequent rows, the predictor variables of each model are shown alongside their standardized β estimates, their unique R^2 (the difference in marginal R^2 between a model including and a model excluding the respective variable), and their structure coefficients $r(\hat{Y},x)$, which represent the correlation between a variable and the fitted model response. In the second to last column, the variation explained by all fixed effects in the model ($R^2_{marginal}$) is shown. The last column shows the variation explained by all fixed effects and phylogenetic relatedness ($R^2_{conditional}$).

Supplementary Note 2 – ABC analysis of postglacial expansion models

Introduction

It is possible that our inference of recent bottlenecks could have been confounded by events in the deeper history of these species. In particular, population sizes during the last glacial maximum (LGM)[20] might have been smaller due to reduced habitat availability or intensified predation. We therefore tested whether small population sizes during the LGM followed by expansions could cause similar genetic patterns across pinnipeds to recent bottlenecks due to anthropogenic exploitation. Specifically, we simulated two additional demographic scenarios that are identical to the bottleneck and non-bottleneck models but which also incorporated a small population size during the LGM and subsequent expansion. We analysed these four models to test the hypotheses that genetic patterns caused by a recent bottleneck can equally be explained by a small population size during the LGM.

Methods

Demographic models. For this supplementary analysis, we explored four different demographic models. These included the two models (bottleneck and non-bottleneck) from the main analysis as well as two additional models that also incorporated a small population size during the LGM and subsequent expansion.

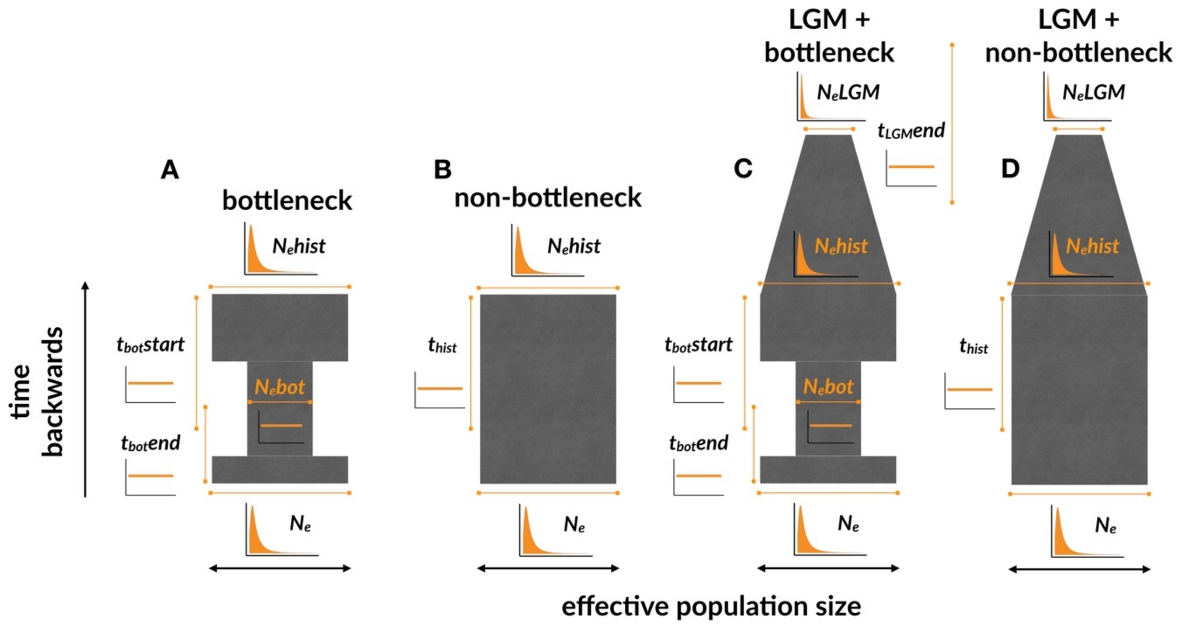

Supplementary Fig. 11: Schematic representation of four contrasting demographic scenarios and the parameter priors defining the models. Panel A depicts the bottleneck model, Panel B the non-bottleneck model, Panel C the LGM + bottleneck model and Panel D the LGM + non-bottleneck model. All priors were drawn independently from each other. For example, the current N_e can be smaller or larger than N_e*hist* for a given species. N_e and N_e*hist* are drawn from the same lognormal distribution while the lognormal prior for N_e*LGM* was defined substantially smaller. Moreover all population size changes across models are defined as instantaneous changes except the transition from N_e*LGM to* N_e*hist* for which an exponential growth rate was

calculated. All prior distributions are also shown as small figures next to the respective parameter. See below for details of the exact priors, calculation of the growth rate and mutation model.

Genetic data under all four models were simulated from broad enough prior distributions to fit all 30 species while keeping the priors as tightly bound as possible around plausible values. The bottleneck model was defined with seven different parameters (Supplementary Fig. 11A). The current effective population size N_e and the historical (i.e. pre-bottleneck) effective population size $N_e hist$ were drawn from a log-normal distribution with N_e ~ lognorm[logmean = 10.5, logsd = 1] and $N_e hist$ ~ lognorm[logmean = 10.5, logsd = 1]. This concentrated sampling within plausible ranges that fitted most species (i.e. with effective population sizes ranging from thousands to tens of thousands of individuals) while also occasionally drawing samples in the hundreds of thousands to fit the few species with very large populations. The bottleneck effective population size $N_e bot$ was drawn from a uniform distribution between 1 and 500 ($N_e bot$ ~ U[1, 500]) while the bottleneck start and end times $t_{bot}start$ and $t_{bot}end$ were drawn from uniform distributions ranging between ten and 70 ($t_{bot}start$ ~U[10, 70]) and one and 30 ($t_{bot}end$ ~ U[1, 30]) generations ago respectively. Hence, the bottleneck time priors encompassed the last four centuries for all species, as their estimated generation times vary between approximately 7 and 19 years (Supplementary Table 1). The microsatellite mutation rate μ was refined after initial exploration and drawn from a uniform prior with μ ~ U[10^{-5}, 10^{-4}] which lies within the range of current empirical estimates [21,22]. The mutation model was defined as a generalized stepwise mutation model with the geometric parameter GSM_{par} reflecting the proportion of multistep mutations, uniformly distributed from GSM_{par} ~U[0, 0.3].

The non-bottleneck model was defined with five parameters (Supplementary Fig. 11B). N_e, $N_e hist$, μ and GSM_{par} were specified with the same priors as previously defined for the bottleneck model and the time parameter corresponding to the historical population size t_{hist} was drawn from a uniform distribution ranging between 10 and 70 generations ago (t_{hist} ~U[10, 70]).

The LGM + bottleneck model was defined with nine parameters (Supplementary Fig. 11C). N_e, $N_e hist$, $N_e bot$, $t_{bot}start$, $t_{bot}end$, μ and GSM_{par} were specified with the same priors as previously described for the bottleneck model. The time parameter for end of the LGM $t_{LGM}end$ was drawn from a uniform distribution ranging between 700 and 1500 generations ago ($t_{LGM}end$ ~U[700, 1500]) and the LGM population size was specified with a log-normal distribution with $N_e LGM$ ~ lognorm[logmean = 9.5, logsd = 1] and was therefore simulated around two-thirds smaller than $N_e hist$. For every simulation, $N_e LGM$ was forced to be smaller than $N_e hist$ to incorporate post-glacial expansion. Moreover, the growth rate between $N_e LGM$ and $N_e hist$ was calculated using these two parameters based on the formula $N_e LGM = N_e hist\ e^{it}$ where i is the growth rate and t is the number of generations of growth between $N_e LGM$ and $N_e hist$.

Finally, the LGM + non-bottleneck model was defined with seven parameters (Supplementary Fig 11D). N_e, $N_e hist$, t_{hist}, μ, GSM_{par} were specified with the same priors as previously described for the non-bottleneck model, while $N_e LGM$ and $t_{LGM} end$ were specified as described above for the LGM + bottleneck model.

ABC analysis. We simulated a total of 2 x 10^6 datasets of 40 individuals and ten microsatellite loci each under the four demographic scenarios using the fastsimcoal function in strataG [23] as an R interface to fastsimcoal2 [24], a continuous-time coalescent simulator. For both the simulated and empirical data, we used five different summary statistics for the ABC inference, all calculated as the mean across loci. Allelic richness (number of alleles), allelic size range, expected heterozygosity (i.e. Nei's gene diversity [25]), the M-ratio [26] and the proportion of low frequency alleles (i.e. with frequencies < 5%). The summary statistics for the empirical datasets were computed by repeatedly re-sampling 40 individuals with replacement from the full datasets and calculating the mean across 1000 subsamples (for the Ladoga ringed seal and the Baltic ringed seal which had sample sizes smaller than 40, the full datasets were taken). As a small number of loci in the empirical data exhibited slight deviations from constant repeat patterns (i.e. not all of the alleles within a locus conformed to a perfect two, three or four bp periodicity), we calculated the M-ratio as an approximation using the most common repeat pattern of a locus to calculate the range of the allele size r and subsequently the M-ratio with $M = k/(r + 1)$ where k is the number of alleles. All statistics were calculated using a combination of functions from the strataG package and self-written code. For the ABC analysis, we used a tolerance threshold of 5 x 10^{-3}, thereby retaining 5000 simulations with summary statistics closest to those of each empirical dataset. For estimating the posterior probability for each scenario and each species, we used the multinomial regression method [27,28] as implemented in the function postpr in the abc package [19] where the model indicator is the response variable of a polychotomous regression and the accepted summary statistics are the predictors.

Results

Model classification evaluation. The confusion matrix visualised in Supplementary Fig. 12 shows the results of the cross-validation evaluation of model classification. Correct rates of model classification were 64% for the bottleneck model, 60% for the LGM + bottleneck model, 69% for the non-bottleneck model and 66% for the LGM + non-bottleneck model. Therefore, specifying the four models broadly enough to fit all 30 species inevitably led to a significant amount of overlap in the simulated genetic diversity, which is reflected in much higher rates of model misclassification than in our main analysis based on two models. This is likely due to the fact that these models produce similar patterns of genetic diversity as many of our summary statistics are known to be sensitive towards recent demographic changes.

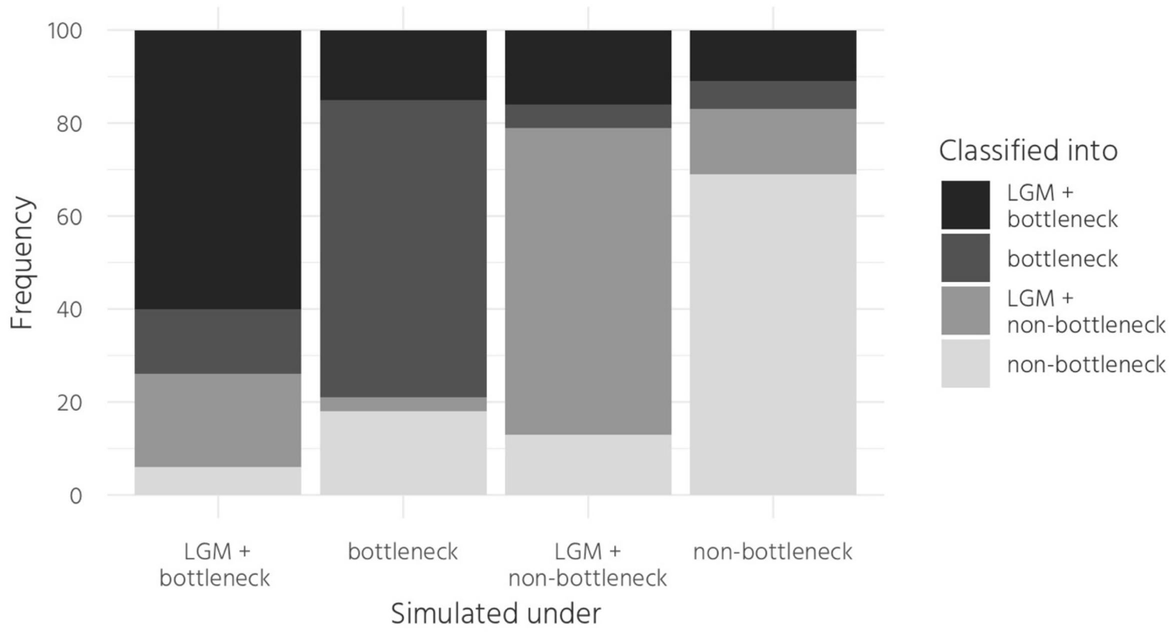

Supplementary Fig. 12: Confusion matrix plot showing misclassification rate estimates from our model selection procedure. Simulations under the four models (LGM + bottleneck, bottleneck, LGM + non-bottleneck, and non-bottleneck) are represented in a sequence from dark to light grey. The four bars represent the model into which a given simulation was classified using ABC.

Model selection. To address our main hypothesis, we evaluated whether recent bottlenecks generate similar patterns of genetic diversity (as measured by our summary statistics) to a small population size during the LGM followed by expansion. Posterior probabilities for the four models are shown for all species in Supplementary Table 11. None of the 11 species that supported the bottleneck model in our main analysis were found to support the LGM + non-bottleneck model in our new analysis. This suggests that genetic patterns in our dataset caused by recent bottlenecks are different from those expected under a postglacial expansion model. Furthermore, in our new analyses all 11 species that originally supported the bottleneck model again had the highest posterior probability for one of the two scenarios incorporating a recent bottleneck. Consequently, for these species, our inference of recent bottlenecks remains unaltered regardless of whether or not these were preceded by an LGM effect.

In the original two-model comparison, the non-bottleneck model was supported for 19 species. Out of these, 14 species again supported a model without a recent bottleneck. Although the remaining five species (Steller sea lion, harbor seal, walrus, South American sea lion and New Zealand sea lion) showed greater support for a model including a recent bottleneck, all of these species had borderline bottleneck model probabilities ($p_{bot} \geq 0.4$) in the original analysis. These slightly different outcomes could potentially be due to lower model classification precision in the four-model analysis. However, they also highlight the importance of interpreting ABC results probabilistically. This is why we used the bottleneck model probability p_{bot} in our main analyses rather than a binary bottleneck / non-bottleneck variable.

**Common name**	**Scientific name**	**LGM + Bottleneck**	**Bottleneck**	**LGM + Non-bottleneck**	**Non-bottleneck**
Weddell Seal	*Leptonychotes weddellii*	0.077	0.079	0.452	0.392
Leopard Seal	*Hydrurga leptonyx*	0.027	0.034	0.533	0.405
Crabeater Seal	*Lobodon carcinophagus*	0.013	0.044	0.219	0.724
Ross Seal	*Ommatophoca rossii*	0.001	0.000	0.992	0.007
Southern Elephant Seal	*Mirounga leonina*	0.217	0.219	0.221	0.343
Northern Elephant Seal	*Mirounga angustirostris*	0.624	0.355	0.019	0.003
Hawaiian Monk Seal	*Monachus schauinslandi*	0.408	0.590	0.002	0.000
Mediterranean Monk Seal	*Monachus monachus*	0.393	0.606	0.002	0.000
Ladoga Ringed Seal	*Phoca hispida ladogensis*	0.295	0.362	0.153	0.190
Saimaa Ringed Seal	*Phoca hispida saimensis*	0.278	0.721	0.001	0.000
Baltic Ringed Seal	*Phoca hispida botnica*	0.164	0.232	0.222	0.382
Ringed Seal	*Pusa hispida*	0.007	0.001	0.122	0.870
Grey Seal	*Halichoerus grypus*	0.305	0.335	0.162	0.198
Harbor Seal	*Phoca vitulina*	0.584	0.097	0.210	0.109
Hooded Seal	*Cystophora cristata*	0.042	0.030	0.700	0.228
Bearded Seal	*Erignathus barbatus*	0.081	0.007	0.827	0.085
Galapagos Fur Seal	*Arctocephalus galapagoensis*	0.298	0.243	0.204	0.254
South American Fur Seal	*Arctocephalus australis*	0.275	0.334	0.154	0.238
New Zealand Fur Seal	*Arctocephalus forsteri*	0.043	0.059	0.354	0.545
Subantarctic Fur Seal	*Arctocephalus tropicalis*	0.055	0.123	0.119	0.703
Antarctic Fur Seal	*Arctocephalus gazella*	0.190	0.571	0.100	0.139
Guadalupe Fur Seal	*Arctocephalus townsendi*	0.314	0.597	0.058	0.032
New Zealand Sea Lion	*Phocarctos hookeri*	0.345	0.161	0.232	0.262
South American Sea Lion	*Otaria byronia / flavescens*	0.309	0.186	0.242	0.263
Australian Fur Seal	*Arctocephalus pusillus*	0.293	0.138	0.336	0.233
Galapagos Sea Lion	*Zalophus wollebaeki*	0.197	0.046	0.588	0.168
California Sea Lion	*Zalophus californianus*	0.296	0.276	0.181	0.246
Steller Sea Lion	*Eumetopias jubatus*	0.396	0.139	0.240	0.225
Northern Fur Seal	*Callorhinus ursinus*	0.021	0.049	0.181	0.748
Walrus	*Odobenus rosmarus*	0.515	0.100	0.223	0.162

Supplementary Table 11: Posterior probabilities of the four models. Shown are the results of the ABC model selection for all 30 species.

Supplementary Note 3–Genetic data collection and genotyping methods

Identification of microsatellite datasets

Separately for each of the 35 extant pinniped species, we conducted Web of Science searches (last updated 28th June 2018) using the search terms described in Supplementary Table 12. In order to maximise the number of recovered records, in each case we combined the term 'microsat*' with all known latin and common species names. We identified a total of 304 unique records (Supplementary Table 12). For each species, we then identified the paper reporting the dataset that was deemed most suitable on the basis of the balance between the number of loci and individuals. As in most cases the raw data were not publically available, we contacted the authors directly to ask for access to the data. Suitable datasets could not be identified or obtained for 13 species. We therefore collated a single suitable microsatellite dataset for each of 25 pinniped species, including three sub-species of ringed seals, and generated new data for a further five species as described below.

Scientific name	Common name	Web of Science search term	Results (n = 304)
Odobenus rosmarus rosmarus	Walrus	("Odobenus rosmarus rosmarus" OR "Walrus") AND microsat*	15
Callorhinus ursinus	Northern Fur Seal	("Callorhinus ursinus" OR "Northern Fur Seal") AND microsat*	4
Neophoca cinerea	Australian Sea Lion	("Neophoca cinerea" OR "Australian Sea Lion") AND microsat*	2
Otaria flavescens	South American Sea Lion	("Otaria flavescens" OR "Otaria byronia" OR "South American Sea Lion") AND microsat*	4
Arctocephalus pusillus doriferus	South African Fur Seal	("Arctocephalus pusillus doriferus" OR "South African Fur Seal" OR "Australian Fur Seal") AND microsat*	1
Phocarctos hookeri	Hookers's Sea Lion	("Phocarctos hookeri" OR "Hookers's Sea Lion" OR "New Zealand Sea Lion") AND microsat*	9
Arctocephalus forsteri	New Zealand Fur Seal	("Arctocephalus forsteri" OR "New Zealand Fur Seal") AND microsat*	6
Arctocephalus australis	South American Fur Seal	("Arctocephalus australis" OR "South American Fur Seal") AND microsat*	6
Arctocephalus galapagoensis	Galapagos Fur Seal	("Arctocephalus galapagoensis" OR "Galapagos Fur Seal") AND microsat*	2
Arctocephalus gazella	Antarctic Fur Seal	("Arctocephalus gazella" OR "Antarctic Fur Seal") AND microsat*	49
Arctocephalus tropicalis	SubAntarctic Fur Seal	("Arctocephalus tropicalis" OR "SubAntarctic Fur Seal") AND microsat*	2
Arctocephalus philippii	Juan Fernandez Fur Seal	("Arctocephalus philippii" OR "Juan Fernandez Fur Seal") AND microsat*	0
Arctocephalus townsendi	Guadalupe Fur Seal	("Arctocephalus townsendi" OR "Guadalupe Fur Seal") AND microsat*	0
Eumetopias jubatus	Steller's Sea Lion	("Eumetopias jubatus" OR "Steller's Sea Lion" OR "Steller Sea Lion") AND microsat*	15
Zalophus californianus	Californian Sea Lion	("Zalophus californianus" OR "Californian Sea Lion") AND microsat*	33
Zalophus wollebaeki	Galapagos Sea Lion	("Zalophus wollebaeki" OR "Galapagos Sea Lion") AND microsat*	10
Erignathus barbatus	Bearded seal	("Erignathus barbatus" OR "Bearded seal") AND microsat*	1
Cystophora cristata	Hooded seal	("Cystophora cristata" OR "Hooded seal") AND microsat*	3
Phoca hispida	Ringed seal	("Phoca hispida" OR "Ringed seal") AND microsat*	13
Phoca sibirica	Baikal seal	("Phoca sibirica" OR "Baikal seal") AND microsat*	0
Halichoerus grypus	Grey seal	("Halichoerus grypus" OR "Grey seal") AND microsat*	52
Phoca caspica	Caspian seal	("Phoca caspica" OR "Caspian seal") AND microsat*	0
Phoca largha	Spotted seal	("Phoca largha" OR "Spotted seal" OR "Largha seal") AND microsat*	2
Phoca vitulina vitulina	Harbour seal	("Phoca vitulina vitulina" OR "Harbour seal") AND microsat*	23
Phoca fasciata	Ribbon seal	("Phoca fasciata" OR "Histriophoca fasciata" OR "Ribbon seal") AND microsat*	0
Phoca groenlandica	Harp seal	("Phoca groenlandica" OR "Harp seal") AND microsat*	4
Lobodon carcinophagus	Crabeater seal	("Lobodon carcinophagus" OR "Crabeater seal") AND microsat*	3
Ommatophoca rossi	Ross seal	("Ommatophoca rossi" OR "Ross seal") AND microsat*	3
Hydrurga leptonyx	Leopard seal	("Hydrurga leptonyx" OR "Leopard seal") AND microsat*	4
Leptonychotes weddelli	Weddell seal	("Leptonychotes weddelli" OR "Weddell seal") AND microsat*	6
Mirounga angustirostris	Northern Elephant seal	("Mirounga angustirostris" OR "Northern Elephant seal") AND microsat*	8
Mirounga leonina	Southern Elephant seal	("Mirounga leonina" OR "Southern Elephant seal") AND microsat*	9
Monachus monachus	Mediterranean Monk seal	("Monachus monachus" OR "Mediterranean Monk seal") AND microsat*	4
Monachus schauinslandi	Hawaiian Monk seal	("Monachus schauinslandi" OR "Hawaiian Monk seal") AND microsat*	8
Monachus tropicalis	Caribbean Monk seal	("Monachus tropicalis" OR "Caribbean Monk seal") AND microsat*	0
Pusa hispida saimensis	Saimaa ringed seal	("Pusa hispida saimensis" OR "Saimaa ringed seal") AND microsat*	3
Pusa hispida ladogensis	Ladoga ringed seal	("Pusa hispida ladogensis" OR "Ladoga ringed seal") AND microsat*	0
Pusa hispida botnica	Baltic ringed seal	("Pusa hispida botnica" OR "Baltic ringed seal") AND microsat*	0

Supplementary Table 12: Identification of microsatellite datasets. We searched relevant papers using scientific names and common names of each species, as shown in the "Web of Science search term" column. The "Results" column shows the number of papers found using the respective search term.

Genotyping methods

Subantarctic fur seal

A total of 88 *Arctocephalus tropicalis* samples were collected from Macquarie Island. Total genomic DNA was extracted from each sample using a standard phenol-chloroform protocol (Sambrook et al 1989) and genotyped at 36 microsatellite loci (see Supplementary Table 13 for details). These were PCR amplified in 5 separate multiplexed reactions using a Type It Kit (Qiagen) as described in Supplementary Table 13. The following PCR profile was used: one cycle of 5 min at 94 °C; 24 cycles of 30 s at 94 °C, 90 s at Ta °C and 30 s at 72 °C; and one final cycle of 15 min at 72 °C (see Supplementary Table 13 for T_a). Fluorescently labelled PCR products were then resolved by electrophoresis on an ABI 3730xl capillary sequencer and allele sizes were scored automatically using GeneMarker v1.95. To ensure high genotype quality, all traces were manually inspected and any incorrect calls were adjusted accordingly.

Locus	Literature source	Multiplex	T_a (°C)
Pv9	Allen et al. [29]	1	53
Hg6.3	Allen et al. [29]	1	53
Hg8.10	Allen et al. [29]	1	53
Hg1.3	Gemmell et al. [30]	1	53
M11a	Hoelzel et al. [31]	1	53
PvcA	Coltman et al. [32]	1	53
ZcwB07	Hoffman et al. [33]	1	53
Agaz2	Hoffman [34]	1	53
Ag3	Hoffman et al. [35]	2	60
Agaz6	Hoffman [34]	2	60
Ag2	Hoffman et al. [35]	2	60
OrrFCB2	Buchanan et al. [36]	2	60
Lw10	Davis et al. [37]	2	60
ZcwC01	Hoffman et al. [33]	2	60
Agaz5	Hoffman [34]	2	60
ZcCgDhB.14	Hernandez-Velazquez et al. [38]	2	60
Ag7	Hoffman et al. [35]	3	60
Agt10	Hoffman and Nichols [39]	3	60
ZcCgDh4.7	Hernandez-Velazquez et al. [38]	3	60
ZcwE05	This study	3	60
Ag1	Hoffman et al. [35]	3	60
OrrFCB8	Buchanan et al. [36]	3	60
Agt47	Hoffman and Nichols [39]	3	60
ZcwF07	Hoffman et al. [33]	4	53
ZcwD02	Wolf et al. [40]	4	53
ZcCgDh1.8	Hernandez-Velazquez et al. [38]	4	53
Aa4	Hoelzel et al. [31]	4	53
ZcCgDh5.8	Hernandez-Velazquez et al. [38]	4	53
Agaz3	Hoffman [34]	4	53
962-1	This study*	5	60
554-6	This study*	5	60

ZcwA12	Hoffman et al. [33]	5	60
PvcE	Coltman et al. [32]	5	60
ZcwB09	Wolf et al. [40]	5	60
Agaz10	Hoffman [34]	5	60
Mang36	Sanvito et al. [41]	5	60

Supplementary Table 13: Microsatellite loci genotyped in the Subantarctic fur seal. "Multiplex" denotes the PCR mastermix into which each locus was multiplexed and "T_a" denotes the annealing temperature used. * Primers (5' to 3') for 962-1 : F-CTACCCCAGGGAGAGTCACT, R-ATACCTGGGCCTCTGGACTT; for 554-6: F-GGCTCCACTTAGCTGGTTGT, R-CCCCTGTTTCATCTTGTGGC)

Northern elephant seal

A total of 260 *Mirounga angustirostris* samples were collected in the southernmost breeding colony of the species, the Islas San Benito (Baja California, Mexico). Total genomic DNA was extracted from each sample using silica-gel membrane technology (DNeasy Blood and Tissue kit, Qiagen; details in Sanvito et al. 2014) and genotyped at 35 microsatellite loci (see Supplementary Table 14 for details). Amplification by PCR was carried out using the "universal tag" method of Schuelke (2000). The microsatellite loci were amplified in singleplex or multiplex reactions as described in Supplementary Table 14. The following PCR profile was used: one cycle of 3 min at 94 °C; 30 cycles of 30 s at 94 °C, 30 s at Ta °C and 40 s at 72 °C; 8 cycles of 30 s at 94 °C, 30 s at 47 °C and 40 s at 72 °C; and one final cycle of 10 min at 72 °C (see Supplementary Table 14 for T_a). Magnesium concentrations varied among the PCR mastermixes as shown in Supplementary Table 14. Fluorescently labelled PCR products were resolved by electrophoresis on an ABI 3730xl capillary sequencer and allele sizes were scored automatically using GeneMarker v1.85. To ensure high genotype quality, all traces were manually inspected and any incorrect calls were adjusted accordingly.

Locus	Literature source	Multiplex	Mg (mM)	T_a (°C)
71HDZ441	Huebinger et al. [42]	–	1.5	54
Hg4.2	Allen et al. [29]	–	1.5	56
Hg8.9	Allen et al. [29]	–	2	48
Lw-16	Davis et al. [37]	–	1.5	55
Lw-20	Davis et al. [37]	–	1.5	49
Lw-8	Davis et al. [37]	–	1.5	47
PVC26	Coltman et al. [32]	–	2	40
PVC74	Coltman et al. [32]	–	2	53
ZcCgDh4.7	Hernandez-Velazquez et al. [38]	–	1.75	56
ZcCgDh7tg	Hernandez-Velazquez et al. [38]	–	2	46
ZcwC03	Wolf et al. [40]	–	1.5	56
ZcwE03	Wolf et al. [40]	–	1.5	54
Hg1.4	Gemmel et al. [30]	1	1.5	53
Lw-18	Davis et al. [37]	1	1.5	53
BG	Gemmell et al [30]	2	2	53
PV9	Goodman et al [43]	2	2	53
Hg3.6	Allen et al. [29]	3	1.75	56
Hg8.10	Allen et al. [29]	3	1.75	56
Hl10	Gelatt et al. (2010)	4	2	39
ZzCgDh3.6	Hernandez-Velazquez et al. [38]	4	2	39
Hg2.3	Garza [44]	5	2	53
Hl-8	Davis et al. [37]	5	2	53
MA11A	Gemmell et al. [30]	5	2	53
CORT	Garza [44]	6	1.75	51
PVC43	Garza [44]	6	1.75	51
Lw-10	Davis et al. [37]	7	1.5	52
PVC1	Garza [44]	7	1.5	52
71HDZ301	Huebinger et al. [42]	8	1.5	42
ZzCgDh1.8	Hernandez-Velazquez et al. [38]	8	1.5	42
ZcwA12	Hoffman et al. [33]	9	1.75	49
ZcwF07	Hoffman et al. [33]	9	1.75	49
Ag-9	Hoffman et al. [35]	10	2	57
ZcwC01	Hoffman et al. [33]	10	2	57

ZcwE04	Hoffman et al. [33]	11	2	52
ZcwG04	Hoffman et al. [33]	11	2	52

Supplementary Table 14: Microsatellite loci genotyped in the Northern elephant seal. "Multiplex" denotes the PCR mastermix into which each locus was multiplexed, "Mg" denotes the concentration of magnesium used in the PCR mastermix and "T_a" denotes the annealing temperature used. Loci not assigned to PCR multiplexes were amplified individually.

Southern elephant seal

A total of 260 *Mirounga leonina* samples were collected at Sea Lion Island, the main breeding colony of the species in the Falkland Islands. Total genomic DNA was extracted from each sample using silica-gel membrane technology (DNeasy Blood and Tissue kit, Qiagen; details in Sanvito et al. 2014) and genotyped at 13 microsatellite loci (see Supplementary Table 15 for details). Amplification by PCR was carried out using the "universal tag" method of Schuelke (2000). The microsatellite loci were amplified in singleplex or multiplex reactions as described in Supplementary Table 15. The following PCR profile was used: one cycle of 3 min at 94 °C; 30 cycles of 30 s at 94 °C, 30 s at Ta °C and 40 s at 72 °C; 8 cycles of 30 s at 94 °C, 30 s at 47 °C and 40 s at 72 °C; and one final cycle of 10 min at 72 °C (see Supplementary Table 15 for T_a). Magnesium concentrations varied among the PCR mastermixes as shown in Supplementary Table 15. Fluorescently labelled PCR products were then resolved by electrophoresis on an ABI 3730xl capillary sequencer and allele sizes were scored automatically using GeneMarker v1.85. To ensure high genotype quality, all traces were manually inspected and any incorrect calls were adjusted accordingly.

Locus	Literature source	Multiplex	Mg (mM)	T_a (°C)
ZcwG04	Hoffman et al. [33]		1.5	54
Lw-20	Davis et al. [37]	1	2	49
OrrFCB9	Buchanan et al. [36]	1	2	49
71HDZ441	Huebinger et al. [42]	2	1.8	56
Ag-8	Hoffman et al. [35]	2	1.8	56
Hg3.6	Allen et al. [29]	3	1.75	58
Hg8.10	Allen et al. [29]	3	1.75	58
ZcwA12	Hoffman et al. [33]	4	2	54
ZcwF07	Hoffman et al. [33]	4	2	54
71HDZ301	Huebinger et al. [42]	5	1.5	42
ZzCgDh1.8	Hernandez-Velazquez et al. [38]	5	1.5	42
ZcCgDh4.7	Hernandez-Velazquez et al. [38]	6	1.9	48
ZcwC01	Hoffman et al. [33]	6	1.9	48

Supplementary Table 15: Microsatellite loci genotyped in the Southern elephant seal. "Multiplex" denotes the PCR mastermix into which each locus was multiplexed, "Mg" denotes the concentration of magnesium used in the PCR mastermix and "T_a" denotes the annealing temperature used. Loci not assigned to PCR multiplexes were amplified individually.

Guadalupe fur seal

A total of 224 *Arctocephalus townsendii* samples were collected from pups of the main breeding colony of the species, Isla Guadalupe (Baja California, Mexico). Total genomic DNA was extracted from each sample using silica-gel membrane technology (DNeasy Blood and Tissue kit, Qiagen; details in Sanvito et al. 2014) and genotyped at 15 microsatellite loci (see Supplementary Table 16 for details). Amplification by PCR was carried out using the "universal tag" method of Schuelke (2000). The following PCR profile was used: one cycle of 3 min at 94 °C; 30 cycles of 30 s at 94 °C, 30 s at Ta °C and 40 s at 72 °C; 8 cycles of 30 s at 94 °C, 30 s at 47 °C and 40 s at 72 °C; and one final cycle of 10 min at 72 °C (see Supplementary Table 16 for T_a). Magnesium concentration used in the PCR mastermix was different for the different primers, as detailed in Supplementary Table 16. Fluorescently labelled PCR products were resolved by electrophoresis on an ABI 3730xl capillary sequencer and allele sizes were scored automatically using GeneMarker v1.85. To ensure high genotype quality, all traces were manually inspected and any incorrect call was adjusted accordingly.

Locus	Literature source	Mg (mM)	T_a (°C)
71HDZ2x	Huebinger et al. [33]	1	45
71HDZ301	Huebinger et al. [33]	2	57
71HDZ441	Huebinger et al. [33]	1.5	56
71HDZ5A	Huebinger et al. [33]	1.5	56
71HDZ5x	Huebinger et al. [33]	1.5	50
Ag-10	Hoffman et al. [35]	1.5	56
Ag-4	Hoffman et al. [35]	1.75	54
Ag-7	Hoffman et al. [35]	1.5	56
ZcCgDh7tg	Hernandez-Velazquez et al. [38]	2	54
ZcwA05	Hoffman et al. [33]	1.5	53
ZcwA12	Hoffman et al. [33]	2	54
ZcwE03	Wolf et al. [40]	1.5	56
ZcwE12	Hoffman et al. [33]	1.5	54
ZcwG04	Hoffman et al. [33]	1.5	53
ZzCgDh5.8	Hernandez-Velazquez et al. [38]	1	47

Supplementary Table 16: Mircosatellite loci genotyped in the Guadalupe fur seal. "Mg" denotes the concentration of magnesium used in the PCR mastermix and "T_a" denotes the annealing temperature used.

Galápagos sea lion

A total of 781 samples were collected from *Zalophus wollebaeki* pups as part of a long-term study on the Galápagos islet of Caamaño (0.45_S, 90.16_W) during 2003–2010 inclusive [45]. Small skin samples were obtained during capture under permission of the Galápagos National Park (PC-001-03 Ext 01, 02, 03-06, 06-08 and PC-043-09). Tissue was stored in 100% ethanol and DNA was subsequently extracted using a DNeasy® tissue kit from Qiagen™. 22 microsatellite loci were PCR amplified and genotyped in four multiplex reactions on an ABI 3730Xl capillary sequencer as specified in Supplementary Table 17 using the Qiagen™ Multiplex PCR kit (for details see Wolf et al. [40] and Hoffman et al. [33]). Genotypes were scored automatically with the MegaBACE® Genetic Profiler and GeneMarker software. To ensure consistency and high quality of genotypes, replicate samples were included on each 96 well plate and all traces were manually curated. Subsets of the data were used in previous studies [46–50].

Locus	Literature source	Multiplex	T_a (°C)
ZcwA05	Hoffman et al. [33]	1	60
ZcwA12	Hoffman et al. [33]	1	60
ZcwD01	Wolf et al. [40]	1	60
ZcwE05	Wolf et al. [46]	1	60
Hg4.2.	Allen et al. [29]	1	60
SGPv9	Allen et al. [29]	1	60
ZcwA07	Wolf et al. [40]	2	60
ZcwB09	Wolf et al. [40]	2	60
ZcwC03	Wolf et al. [40]	2	60
ZcwC11	Wolf et al. [40]	2	60
ZcwD02	Wolf et al. [40]	2	60
ZcwH09	Wolf et al. [40]	2	60
ZcCgDh5.8	Hernandez-Velazquez et al. [51]	2	60
ZcwE03	Wolf et al. [40]	3	60
ZcwF07	Hoffman et al. [33]	3	60
Hg6.1	Allen et al. [29]	3	60
Hg8.10	Allen et al. [29]	3	60
ZcCgDh7tg	Hernandez-Velazquez et al. [51]	3	60
ZcwB07	Hoffman et al. [33]	4	60
ZcwE04	Hoffman et al. [33]	4	60
ZcwE12	Hoffman et al. [33]	4	60
SGPv11	Goodman SJ [52]	4	60

Supplementary Table 17: Microsatellite loci genotyped in the Galápagos sea lion. "Multiplex" denotes the PCR mastermix into which each locus was multiplexed and "T_a" denotes the annealing temperature used.

Supplementary References

1. Wilke, C. O. Ridgeline Plots in 'ggplot2' [R package ggridges version 0.4.1].
2. Krüger, O., Wolf, J. B. W., Jonker, R. M., Hoffman, J. I. & Trillmich, F. Disentangling the contribution of sexual selection and ecology to the evolution of size dimorphism in pinnipeds. *Evolution* **68,** 1485–1496 (2014).
3. The IUCN Red List of Threatened Species. **Version 2017-3.,** (2017).
4. Davis, C. S., Stirling, I., Strobeck, C. & Coltman, D. W. Population structure of ice-breeding seals. *Mol. Ecol.* **17,** 3078–3094 (2008).
5. Schultz, J. K., Baker, J. D., Toonen, R. J., Harting, A. L. & Bowen, B. W. Range-wide genetic connectivity of the Hawaiian monk seal and implications for translocation. *Conserv. Biol.* **25,** 124–132 (2010).
6. Pastor, T., Garza, J. C., Allen, P., Amos, W. & Aguilar, A. Low genetic variability in the highly endangered mediterranean monk seal. *J. Hered.* **95,** 291–300 (2004).
7. Nyman, T. *et al.* Demographic histories and genetic diversities of Fennoscandian marine and landlocked ringed seal subspecies. *Ecol. Evol.* **4,** 3420–3434 (2014).
8. Klimova, A. *et al.* Global population structure and demographic history of the grey seal. *Mol. Ecol.* **23,** 3999–4017 (2014).
9. Rijks, J. M., Hoffman, J. I., Kuiken, T., Osterhaus, A. & Amos, W. Heterozygosity and lungworm burden in harbour seals (Phoca vitulina). *Heredity* **100,** 587 (2008).
10. Coltman, D. W. *et al.* Panmictic population structure in the hooded seal (Cystophora cristata). *Mol. Ecol.* **16,** 1639–1648 (2007).
11. Lopes, F. *et al.* Fine-scale matrilineal population structure in the Galapagos fur seal and its implications for conservation management. *Conserv. Genet.* **16,** 1099–1113 (2015).
12. de Oliveira, L. R. *et al.* Morphological and genetic evidence for two evolutionarily significant units (ESUs) in the South American fur seal, Arctocephalus australis. *Conserv. Genet.* **9,** 1451–1466 (2008).
13. Dussex, N. *et al.* Low spatial genetic differentiation associated with rapid recolonization in the New Zealand Fur Seal Arctocephalus forsteri. *J. Hered.* **107,** 581–592 (2016).
14. Hoffman, J. I., Grant, S. M., Forcada, J. & Phillips, C. D. Bayesian inference of a historical bottleneck in a heavily exploited marine mammal. *Mol. Ecol.* **20,** 3989–4008 (2011).
15. Osborne, A. J. *et al.* Genetic Evidence of a Population Bottleneck and Inbreeding in the Endangered New Zealand Sea Lion, Phocarctos hookeri. *J. Hered.* **107,** esw015–402 (2016).
16. Hoffman, J. I. *et al.* Population structure and historical demography of South American sea lions provide insights into the catastrophic decline of a marine mammal population. *R. Soc. Open Sci.* **3,** (2016).
17. Dickerson, B. R., Ream, R. R., Vignieri, S. N. & Bentzen, P. Population structure as revealed by mtDNA and microsatellites in northern fur seals, Callorhinus ursinus, throughout their range. *PLoS One* **5,** e10671 (2010).

18. Shafer, A. B., Davis, C. S., Coltman, D. W. & Stewart, R. E. Microsatellite assessment of walrus (Odobenus rosmarus rosmarus) stocks in Canada. *NAMMCO Sci. Publ.* **9,** 15–31 (2014).
19. Csilléry, K., François, O. & Blum, M. G. B. abc: an R package for approximate Bayesian computation (ABC). *Methods Ecol. Evol.* **3,** 475–479 (2012).
20. Clark, P. U. *et al.* The last glacial maximum. *Science* **325,** 710–714 (2009).
21. Ellegren, H. Microsatellites: simple sequences with complex evolution. *Nat. Rev. Genet.* **5,** 435–445 (2004).
22. Selkoe, K. A. & Toonen, R. J. Microsatellites for ecologists: a practical guide to using and evaluating microsatellite markers. *Ecol. Lett.* **9,** 615–629 (2006).
23. Archer, F. I., Adams, P. E. & Schneiders, B. B. stratag: An r package for manipulating, summarizing and analysing population genetic data. *Mol. Ecol. Resour.* **17,** 5–11 (2017).
24. Excoffier, L., Dupanloup, I., Huerta-Sánchez, E., Sousa, V. C. & Foll, M. Robust demographic inference from genomic and SNP data. *PLoS Genet.* **9,** e1003905 (2013).
25. Nei, M. Analysis of gene diversity in subdivided populations. *Proc. Natl. Acad. Sci.* **70,** 3321–3323 (1973).
26. Garza, J. C. & Williamson, E. G. Detection of reduction in population size using data from microsatellite loci. *Mol. Ecol.* **10,** 305–318 (2001).
27. Beaumont, M. A., Zhang, W. & Balding, D. J. Approximate Bayesian computation in population genetics. **162,** 2025–2035 (2002).
28. Fagundes, N. J. *et al.* Statistical evaluation of alternative models of human evolution. *Proc. Natl. Acad. Sci.* **104,** 17614–17619 (2007).
29. Allen, P. J., Amos, W., Pomeroy, P. P. & Twiss, S. D. Microsatellite variation in grey seals (Halichoerus grypus) shows evidence of genetic differentiation between two British breeding colonies. *Mol. Ecol.* **4,** 653–662 (1995).
30. Gemmell, N. J., Allen, P. J., Goodman, S. J. & Reed, J. Z. Interspecific microsatellite markers for the study of pinniped populations. *Mol. Ecol.* **6,** 661–666 (1997).
31. Hoelzel, A. R., Le Boeuf, B. J., Reiter, J. & Campagna, C. Alpha-male paternity in elephant seals. *Behav. Ecol. Sociobiol.* **46,** 298–306 (1999).
32. Coltman, D. W., Bowen, W. D. & Wright, J. M. PCR primers for harbour seal (Phoca vitulina concolour) microsatellites amplify polymorphic loci in other pinniped species. *Mol. Ecol.* **5,** 161–163 (1996).
33. Hoffman, J. I., Steinfartz, S. & Wolf, J. B. W. Ten novel dinucleotide microsatellite loci cloned from the Galápagos sea lion (Zalophus californianus wollebaeki) are polymorphic in other pinniped species. *Mol. Ecol. Notes* **7,** 103–105 (2007).
34. Hoffman, J. I. A panel of new microsatellite loci for genetic studies of antarctic fur seals and other otariids. *Conserv. Genet.* **10,** 989–992 (2009).
35. Hoffman, J. I., Dasmahapatra, K. K. & Nichols, H. J. Ten novel polymorphic dinucleotide microsatellite loci cloned from the Antarctic fur seal Arctocephalus gazella. *Mol. Ecol. Resour.* **8,** 459–461 (2008).

36. Buchanan, F. C., Maiers, L. D., Thue, T. D., De March, B. G. & Stewart, R. E. Microsatellites from the Atlantic walrus Odobenus rosmarus rosmarus. *Mol. Ecol.* **7,** 1083–1085 (1998).
37. Davis, C. S., Gelatt, T. S., Siniff, D. & Strobeck, C. Dinucleotide microsatellite markers from the Antarctic seals and their use in other Pinnipeds. *Mol. Ecol. Notes* **2,** 203–208 (2002).
38. Hernandez-Velazquez, F. D. *et al.* New polymorphic microsatellite markers for California sea lions (Zalophus californianus). *Mol. Ecol. Notes* **5,** 140–142 (2005).
39. Hoffman, J. I. & Nichols, H. J. A Novel Approach for mining polymorphic microsatellite markers in silico. *PLOS ONE* **6,** e23283 (2011).
40. Wolf, J. B. W., Tautz, D., Caccone, A. & Steinfartz, S. Development of new microsatellite loci and evaluation of loci from otherpinniped species for the Galápagos sea lion (Zalophus californianus wollebaeki). *Conserv. Genet.* **7,** 461–465 (2006).
41. Sanvito, S. *et al.* Isolation and cross-species amplification of novel microsatellite loci in a charismatic marine mammal species, the northern elephant seal (Mirounga angustirostris). *Conserv. Genet. Resour.* **5,** 93–96 (2013).
42. Huebinger, R. M., Louis Jr, E. E., Gelatt, T., Rea, L. D. & Bickham, J. W. Characterization of eight microsatellite loci in Steller sea lions (Eumetopias jubatus). *Mol. Ecol. Notes* **7,** 1097–1099 (2007).
43. Goodman, S. J. Dinucleotide repeat polymorphisms at seven anonymous microsatellite loci cloned from the European harbour seal (Phoca vitulina vitulina). *Anim. Genet.* **28,** 310–311 (1997).
44. Garza, J. C. Population genetics of the northern elephant seal. (2000).
45. Wolf, J. B. W., Kauermann, G. & Trillmich, F. Males in the shade: habitat use and sexual segregation in the Galápagos sea lion (Zalophus californianus wollebaeki). *Behav. Ecol. Sociobiol.* **59,** 293–302 (2005).
46. Wolf, J. B. W., Tautz, D. & Trillmich, F. Galápagos and Californian sea lions are separate species: genetic analysis of the genus Zalophus and its implications for conservation management. *Front. Zool.* **4:20,** 1–13 (2007).
47. Wolf, J. B. W. *et al.* Tracing early stages of species differentiation: Ecological, morphological and genetic divergence of Galapagos sea lion populations. *BMC Evol. Biol.* **8,** 150 (2008).
48. Wolf, J. B. W. & Trillmich, F. Kin in space. Social viscosity in a spatially and genetically sub-structured network. *Proc. R. Soc. B-Biol. Sci.* **275,** 2063–2069 (2008).
49. Pörschmann, U., Trillmich, F., Müller, B. & Wolf, J. B. W. Male reproductive success and its behavioural correlates in a polygynous mammal, the Galápagos sea lion (Zalophus wollebaeki). *Mol. Ecol.* **19,** 2574–2586 (2010).
50. Lenz, T. L., Mueller, B., Trillmich, F. & Wolf, J. B. W. Divergent allele advantage at MHC-DRB through direct and maternal genotypic effects and its consequences for allele pool composition and mating. *Proc. R. Soc. B Biol. Sci.* **280,** 20130714 (2013).
51. Hernandez-Velazquez, F. D. *et al.* New polymorphic microsatellite markers for California sea lions (Zalophus californianus). *Mol. Ecol. Notes* **5,** 140–142 (2005).

52. Goodman, S. J. Dinucleotide repeat polymorphisms at seven anonymous microsatellite loci cloned from the European Harbour Seal (Phoca vitulina vitulina). *Anim. Genet.* **28,** 310–311 (1997).
